# Transcriptomic profiling analysis to identify genes associated with PA biosynthesis and insolubilization in the late stage of fruit development in C-PCNA persimmon

**DOI:** 10.1038/s41598-022-23742-4

**Published:** 2022-11-09

**Authors:** Yiru Wang, Qi Zhang, Tingting Pu, Yujing Suo, Weijuan Han, Songfeng Diao, Huawei Li, Peng Sun, Jianmin Fu

**Affiliations:** 1 Key Laboratory of Non-Timber Forest Germplasm Enhancement and Utilization of State Administration of Forestry and Grassland, Research Institute of Non-Timber Forestry, Chinese Academy of Forestry, No. 3 Weiwu Road, Jinshui District, Zhengzhou, 450003 China; 2 College of Forestry, Inner Mongolia Agricultural University, Hohhot, 010018 China

**Keywords:** Plant sciences, Plant breeding, Plant cell biology, Plant physiology, Secondary metabolism

## Abstract

PA-enhanced content causes astringency in persimmon fruit. PCNA persimmons can lose their astringency naturally and they become edible when still on the tree, which allows for conserves of physical and financial resources. C-PCNA persimmon originates in China. Its deastringency trait primarily depends on decreased PA biosynthesis and PA insolubilization at the late stage of fruit development. Although some genes and transcription factors that may be involved in the deastringency of C-PCNA persimmon have been reported, the expression patterns of these genes during the key deastringency stage are reported less. To investigate the variation in PA contents and the expression patterns of deastringency-related genes during typical C-PCNA persimmon ‘Xiaoguo-tianshi’ fruit development and ripening, PA content and transcriptional profiling were carried out at five late stages from 70 to 160 DAF. The combinational analysis phenotype, PA content, and DEG enrichment revealed that 120–140 DAF and 140–160 DAF were the critical phases for PA biosynthesis reduction and PA insolubilization, respectively. The expression of PA biosynthesis-associated genes indicated that the downregulation of the *ANR* gene at 140–160 DAF may be associated with PA biosynthesis and is decreased by inhibiting its precursor cis-flavan-3-ols. We also found that a decrease in acetaldehyde metabolism-associated *ALDH* genes and an increase in *ADH* and *PDC* genes might result in C-PCNA persimmon PA insolubilization. In addition, a few *MYB-bHLH-WD40* (MBW) homologous transcription factors in persimmon might play important roles in persimmon PA accumulation. Furthermore, combined coexpression network analysis and phylogenetic analysis of MBW suggested that three putative transcription factors *WD40* (*evm.TU.contig1.155*), *MYB* (*evm.TU.contig8910.486*) and *bHLH* (*evm.TU.contig1398.203*), might connect and co-regulate both PA biosynthesis and its insolubilization in C-PCNA persimmon. The present study elucidated transcriptional insights into PA biosynthesis and insolubilization during the late development stages based on the C-PCNA *D. kaki* genome (unpublished). Thus, we focused on PA content variation and the expression patterns of genes involved in PA biosynthesis and insolubilization. Our work has provided additional evidence on previous knowledge and a basis for further exploration of the natural deastringency of C-PCNA persimmon.

## Introduction

*Diospyros kaki* Thunb. is a member of the family Ebenaceae and is among the important fruit trees cultivated and harvested worldwide^1^. Based on genetic traits and astringency removal, persimmon varieties can be classified into pollination-constant non-astringent (PCNA), pollination-variant non-astringent (PVNA), pollination-variant astringent (PVA), and pollination-constant astringent (PCA) types^2^. The Chinese PCNA (C-PCNA) and Japanese PCNA (J-PCNA) persimmon can lose astringency naturally on the tree without any postharvest treatment but have different proanthocyanidin (PA) accumulation mechanisms^3,4^. The characteristic of losing astringency in J-PCNA and C-PCNA persimmon is genetically controlled by a recessive and a dominant allele, respectively^5^. Because of dominant inheritance, the C-PCNA genotype is often selected as the superior parent for persimmon breeding programs.

Both the “dilution effect” and “coagulation effect” contribute to the deastringency of C-PCNA persimmon fruit at the late stage^6^. The “dilution effect” means that PA biosynthesis is reduced with the fruit grows larger, resulting in a decrease in the proportion of soluble PA content in persimmon fruit^7,8^. PAs are synthesized via the chorismic acid pathway, phenylpropane metabolic pathway, flavonoid synthesis and proanthocyanidin pathway^9^. The structural genes encoding the enzymes have been identified in the three pathways, including chalcone isomerase (*CHI*), chalcone synthase (*CHS*), leucoanthocyanidin reductase (*LAR*), flavonoid 3′-hydroxylase (*F3′H*), and anthocyanidin reductase (*ANR*)^10^. The “coagulation effect” refers to losing astringency by PA insolubilization by converting soluble PAs into insoluble PAs with acetaldehyde^6^. The acetaldehyde metabolic pathway plays an important role in the astringency loss of PCNA persimmon^11,12^ and is catalyzed by pyruvate decarboxylase (*PDC*), alcohol dehydrogenase (*ADH*), and acetaldehyde dehydrogenase (*ALDH*)^13,14^.

Transcription factors involved in PA biosynthesis have been identified previously. The *R2R3MYB*, *bHLH*, and *WD40* (MBW) complex regulates the expression of the structural genes *DFR*, *ANS*, and *ANR* of late flavonoid/phenylpropanoid metabolism^15^. The expression of PA biosynthesis genes is specifically induced by *AtTT2* (*AtMYB123*), *AtTT8* (*AtbHLH042*), and *AtTTG1* (*WD40-repeat* protein) in *Arabidopsis thaliana*^16^. In strawberries, *FaTTG1* can form a complex with *FaMYB9*/*FaMYB11* and *FabHLH3*, which increases the accumulation of PA by upregulating the expression of *ANS* and *LAR*^17^. In persimmon, reduced expression of *DkMYB4* in J-PCNA leads to the downregulation of PA biosynthesis during the early stages^18^, and in C-PCNA, reduced expression of *DkMYB14* results in the nonastringency, which directly represses PA biosynthesis and promotes its insolubilization, leading to nonastringency^19^. A *bHLH* gene (*DkMYC1*) involved in PA synthesis regulation was isolated from ‘Luotian-tianshi’, and the expression pattern of *DkMYC1* was correlated with PA accumulation and the expression patterns of *DkANR* and *DkF3′5′H*^20^.

Several studies have tried to elucidate the underlying molecular basis for C-PCNA persimmon deastringency, either by ethanol or by 40 °C water treatments via transcriptome sequencing. These procedures have no doubt provided crucial insight into the deastringency process^21,22^; however, C-PCNA persimmon can lose its astringency naturally. The PA biosynthesis and insolubilization in C-PCNA persimmon have not been adequately explained in the natural state, especially at the late developmental stages of fruit. In this study, fruits from the C-PCNA genotype ‘Xiaoguo-tianshi’ persimmon were specifically collected at five later stages, as natural deastringency occurs late on the C-PCNA persimmon tree. In addition, the completion of the C-PCNA *D. kaki* reference genome (unpublished) has facilitated the genome-wide analysis of dynamic gene expression during fruit development; thus, the PA content combined with RNA-seq analysis at key late development stages can provide basic support for C-PCNA persimmon natural astringency loss.

## Material and methods

### Plant material

Fruits of C-PCNA persimmon (*D.kaki*, variety ‘Xiaoguo-tianshi’) at five developmental stages (T1 = 70, T2 = 100, T3 = 120, T4 = 140, T5 = 160 DAF) were harvested from Yuanyang County, Henan Province, China (34° 55′ 18″–34° 56′ 27″ N, 113° 46′ 14″–113° 47′ 35″ E). The collection and conservation of plant material in this study complied with relevant institutional, national, and international guidelines and legislation. Fresh material was immediately frozen in liquid nitrogen and stored at − 80 °C until the PA content and RNA extraction were analyzed.

### Extraction and determination of PAs

Approximately 2.5 g (precise weight) of the persimmon fruit powder was extracted with 80% (v/v) ethanol in an ultrasonic bath for 30 min, and the precipitates were suspended in 1% HCl-MeOH for insoluble PA extraction. Soluble and insoluble PA contents were detected using the Folin-Ciocalteu method as described by Oshida et al.^23^.

### RNA extraction, transcriptome sequencing, and data analysis

Total RNA was extracted using the UNlQ-10 Column TRIzol Total RNA Isolation Kit (B511321; Sangon, Shanghai, China). The quality and quantity of the RNA were tested on a 1% agarose gel and then assessed on a Merinton SMA4000 spectrophotometer (Merinton Inc., Beijing, China). The paired-end cDNA sequencing libraries of fruits at five stages were prepared with three biological replicates and then sequenced on an Illumina NovaSeq platform (Illumina, San Diego, CA, USA). The files of raw reads were cleaned by removing adapter sequences and then the clean reads were mapped to the hexaploid C-PCNA persimmon genome (variety ‘Xiaoguo-tianshi’, unpublished).

DEseq2 was used to detect the DEGs^24^, with a padj ≤ 0.05 and |log2-fold change|≥ 1. Gene Ontology (GO) (www.geneontology.org)^25^ and Kyoto Encyclopedia of Genes and Genomes (KEGG) (www.kegg.jp/kegg/kegg1.html)^26^ databases were used for functional enrichment analysis by ClusterProfiler (3.8.1)^27^. The R packages Cluster, Biobase, and Q-value were used to display the expression profiles of the DEGs in the five stages using K-means clustering. Heatmaps were prepared with the online website Hiplot. Pearson’s correlation analysis was performed using SPSS statistical software (v 24.0; SPSS Inc., Chicago, IL, USA). The coexpression networks were constructed using DEGs and were analyzed using the R package WGCNA^28^ and visualized using Cytoscape (v3.9.1) and its plugin cytoHubba^29^.

### Quantitative RT-PCR analysis

Total RNA for the RNA-seq analysis was reverse-transcribed into cDNA with the TRUE-script First-Strand cDNA Synthesis Kit (Kemix, Beijing, China). Reactions were run on a LightCycler 480 II (Roche) with a 96-well plate. The reaction conditions for each gene were 95 °C for 3 min, followed by 45 cycles of 5 s at 95 °C and 30 s at 55–60 °C. Three technical replicates were performed for each gene. *GAPDH* was used as the reference gene^30^. The RT-qPCR primers are listed in Table S1.

### Identification of *MYB-bHLH-WD40* complex members in C-PCNA persimmon

To identify *MYB-bHLH-WD40* Complex Members in the persimmon genome, local tBLASTp (https://blast.ncbi.nlm.nih.gov/) (E-value ≤ 1e^−5^) was performed using the sequences of homologous MBW complex proteins in *Arabidopsis thaliana*^16,31^, *Malus* × *domestica*^32^, *Vitis vinifera*^33–35^, and *Fragaria* × *ananassa*^17^ (Table S2). The online website Pfam (https://pfam.xfam.org/) was used to remove sequences without conserved MYB_DNA-binding (PF00249.30, PF13921.5), bHLH-MYC_N (PF14215.5), or WD40 (PF00400.31) domains (Table S2). Multiple sequence alignments and phylogenetic analysis were implemented using Clustal X^36^ and MEGA 5^37^ software, respectively.

## Result

### Phenotype and PA content of C-PCNA persimmon fruit

**Figure 1:**
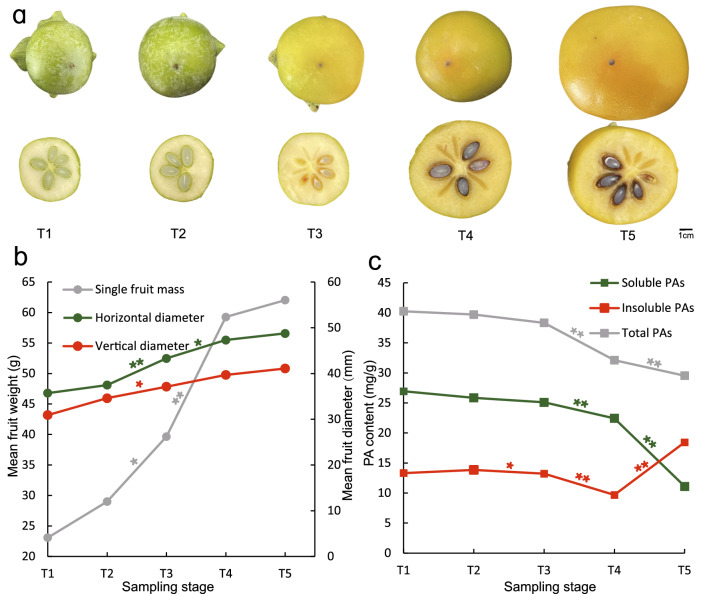
Five developmental stages of C-PCNA persimmon ‘Xiaoguo-tianshi’. (**a**) Fruit morphology. (**b**) Mean fruit weight and diameter. (**c**) Soluble PAs, insoluble PAs, and total PA content. Values are the means of four replicates. **Indicates a significant difference at *P* < 0.01. * indicates a significant difference at *P* < 0.05.

To evaluate the C-PCNA persimmon deastringency process, the fruit’s late developmental stages were grouped into 5 time points (T1–T5) and the fruits were harvested at 70, 100, 120, 140, and 160 days after flower (DAF) (Fig. 1a). Morphological differences were measured based on the changes in fruit size and weight. Persimmon fruit experienced the fastest growth stage from 120 to 140 DAF, with a 39.67–59.27 g weight, and then grew slowly until deastringency (Fig. 1b). During the ‘expansion’ phase, the soluble and insoluble PA contents decreased, followed by a continuous decrease in the soluble PA content in the T4-T5 stages and a significant increase in the insoluble PA content. This resulted in the astringency loss of ‘Xiaoguo-tianshi’ persimmon fruit at stage T5 (Fig. 1c).

### RNA-Seq of C-PCNA persimmon developing fruit

**Figure 2:**
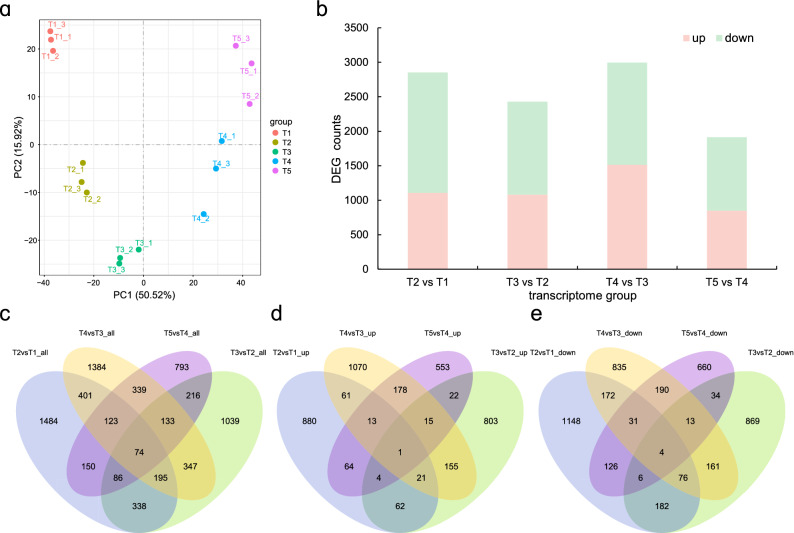
Overview of the C-PCNA Persimmon Developing Fruit transcriptome. (**a**) Principal component analysis. (**b**) Summary of DEGs in all combinations of developmental stage comparisons. The number of total DEGs (**c**), upregulated DEGs (**d**), and downregulated DEGs (**e**) are presented by Venn diagrams (|log2-fold change|> 1 and padj < 0.05).

After the low-quality reads, the transcriptome sequencing of the ‘Xiaoguo-tianshi’ fruit at five developmental stages yielded 99.24 GB of data. Each filtered sample consisted of 6.79 GB of high-quality data with a Q30 base percentage of 95.28% on average, approximately 87.61% of the reads could be with the reference *D. kaki* genome, and 3,472 novel genes were also identified. These results indicated that sequencing data accuracy was sufficient for further analysis. A principal component analysis (PCA) based on Fragments Per Kilobase of transcript per Million mapped reads (FPKM) values separated the samples into five distinct groups, with each sample making a separate group with its replicates, indicating that there were good correlations and differences among the samples (Fig. 2a). To analyze the expression variabilities associated with C-PCNA persimmon fruit PA biosynthesis and insolubilization at different developmental stages, genes in four pairwise transcriptome groups (T2 vs T1, T3 vs T2, T4 vs T3, and T5 vs T4) were compared. In all, 7,102 genes were significantly differentiated in the four groups, including 2,851 DEGs in T2 vs T1, 2,428 DEGs in T3 vs T2, 2,996 DEGs in T4 vs T3, and 1,914 DEGs in T4 vs T3. The Venn diagram shows that 1,484, 1039, 1,384, and 793 DEGs were differentially expressed in the four respective groups (Fig. 2b–e). Most of the DEGs were downregulated in the control groups. The reliability of the transcriptomic results was confirmed by the consistent gene expression trend of 9 DEGs, which was observed using real-time quantitative reverse transcription polymerase chain reaction (RT-qPCR) analysis (Fig. S1) compared with the transcriptome FPKM data.

### Functional enrichment analysis of DEGs

The GO and KEGG databases were used to further analyze DEGs in four comparison groups (T2 vs T1, T3 vs T2, T4 vs T3, and T5 vs T4) using padj < 0.05 to indicate a significant difference. The results of GO functional enrichment analysis revealed significant enrichment in four terms associated with PA transport: UDP-glycosyltransferase, glucosyltransferase, drug transmembrane transporter, and transferase activities. GO terms of glucosyltransferase activity were observed in T2 vs T1, T4 vs T3, and T5 vs T4, and the other three terms were only shared in T5 vs T4. KEGG pathway enrichment analysis revealed that T4 vs T3 had enriched phenylpropanoid biosynthesis. The significantly enriched KEGG pathway term flavonoid biosynthesis was shared in T4 vs T3 and T5 vs T4. The two candidate KEGG pathways were considered to be heavily involved in PA accumulation. Our results suggest that the T3–T4 stages may be a key developmental period for the persimmon PA biosynthesis process phenylpropane metabolic pathway and flavonoid synthesis proanthocyanidin pathway. Stages T4-T5 may be involved in persimmon PA transport and the subsequent process (Fig. S2).

### Gene expression trends during C-PCNA persimmon fruit development

**Figure 3:**
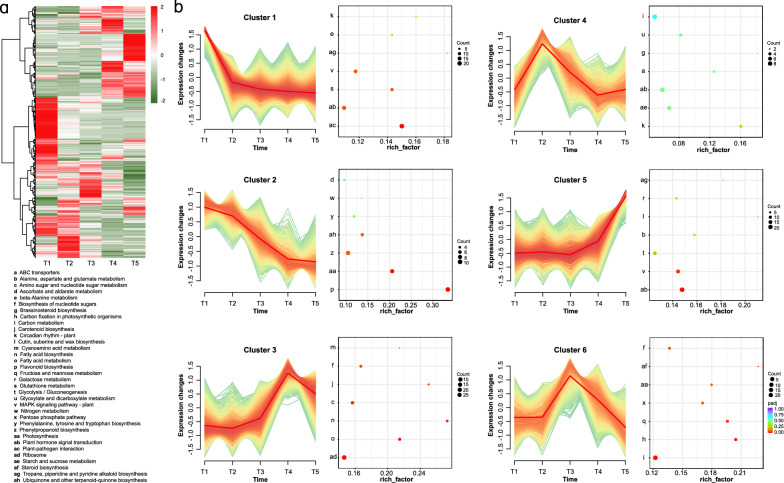
Gene expression profiles and KEGG enrichment analysis of the DEGs in the six common expression clusters composing the fruit transcriptome. (**a**) Heatmap of the overall common expression pattern. Heat maps depict the normalized gene expression values, which represent the mean value of three biological replicates. (**b**) Expression patterns and KEGG pathway annotations of 6 clusters. Each line represents the normalized gene expression values for an individual transcript. The y-axis of each cluster is the pathway name, while the x-axis represents the rich factor. Significantly overrepresented KEGG categories are represented by red dots.

Six gene profiles based on their expression patterns were clustered together, as shown in Fig. 3a. The genes within these 6 expression profiles were analyzed by KEGG annotation (Fig. 3b). Cluster 2 comprised 1,090 DEGs and their expression showed a downregulating trend from T1 to T5, and these genes rapidly decreased between T2 and T4. Cluster 5 contained 1,332 DEGs, and their expression increased at late time points. The expression pattern of cluster 2 was positively consistent with soluble PA and total PA content (Fig. 1b), whereas 5 was positively aligned with insoluble PA content. DEGs in cluster 2 were significantly enriched in flavonoid biosynthesis and photosynthesis, where flavonoid biosynthesis had an enrichment factor of nearly 0.4, indicating greater intensity. The plant hormone signal transduction and MAPK signaling pathways were significantly enriched in cluster 5. Among those, some of the final metabolites of flavonoid biosynthesis are proanthocyanins. The results of the functional classification analysis of the common expression patterns of DEGs combined with KEGG showed that genes in cluster 2 and cluster 5 might be associated with PA accumulation and were selected for subsequent analysis.

### DEGs related to PA biosynthesis and insolubilization

**Figure 4:**
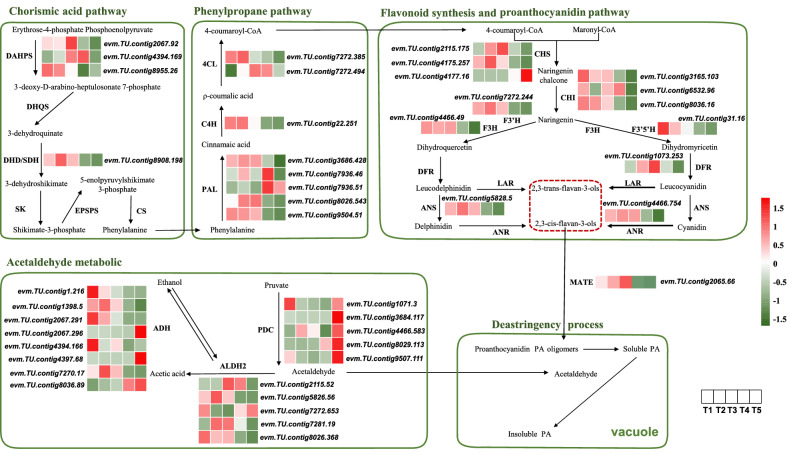
Expression patterns of 43 DEGs involved in PA biosynthesis and insolubilization. PA biosynthesis pathways include the chorismic acid pathway, phenylpropane pathway, flavonoid synthesis and proanthocyanidin-specific pathway. PA insolubilization refers to converting soluble PA into insoluble PA with acetaldehyde metabolism. Heatmaps depict the normalized gene expression values, which represent the mean value of three biological replicates.

PA accumulation gene expression levels across the five developmental stages were investigated by generating heat maps (Fig. 4). We focused on the 43 DEGs involved in the PA biosynthesis and insolubilization pathway, which included 18 genes from cluster 2, 8 from cluster 5, 7 from cluster 6, 5 from cluster 4, 3 from cluster 1, and 2 from cluster 3. Since the aim was to identify genes associated with PA content variation in the C-PCNA persimmon, our analysis was focused on DEGs in clusters 2 and 5. Nearly all DEGs from cluster 2 were downregulated in T4 vs T3 and T5 vs T4. Most of the cluster 2 genes were involved in PA biosynthesis, such as *DAHPS* (*evm.TU.contig8955.26*) and *DHD/SDH* (*evm.TU.contig8908.198*), the precursors of proanthocyanin biosynthesis that participate in the chorismic acid pathway. *PAL* (*evm. TU.contig9504.51, evm.TU.contig3686.428*), *C4H* (*evm.TU.contig22.251*)*,* and *4CL* (*evm.TU.contig7272.385*) are phenylpropanoid genes. *ANR* (*evm.TU.contig4466.754*), *ANS* (*evm.TU.contig5828.5*), *CHS* (*evm.TU.contig4175.257*), *F3’H* (*evm.TU.contig7272.244*), *F3′5'H* (*evm.TU.contig31.16*), and *F3H* (*evm.TU.contig4466.49*) are involved in flavonoid synthesis and the proanthocyanidin pathway. Cluster 5 DEGs, however, were upregulated in T5 vs T4, and most of these genes were involved in PA insolubilization, such as *PDC* (*evm.TU.contig3684.117*, *evm.TU.contig4466.583*, *evm.TU.contig8029.113, evm.TU.contig9507.111*), *ADH* (*evm.TU.contig2067.296*, *evm.TU.contig4397.68*)*,* and *ALDH* (*evm.TU.contig7272.653*), which take part in acetaldehyde metabolism. However, the *MATE* gene (*evm.TU.contig2065.66*) was significantly downregulated at T4 vs T3, which might be involved in transporting PA to the vacuole. This study revealed that PA biosynthesis-associated genes were highly downregulated in T4 vs T3 and T5 vs T4, whereas PA insolubilization genes were highly upregulated in T5 vs T4, providing a reasonable explanation for the variation in PA content at stages T4–T3 and T5–T4.

### Phylogenetic analysis and expression patterns of the persimmon *MYB*-*bHLH*-*WD40* complex

**Figure 5:**
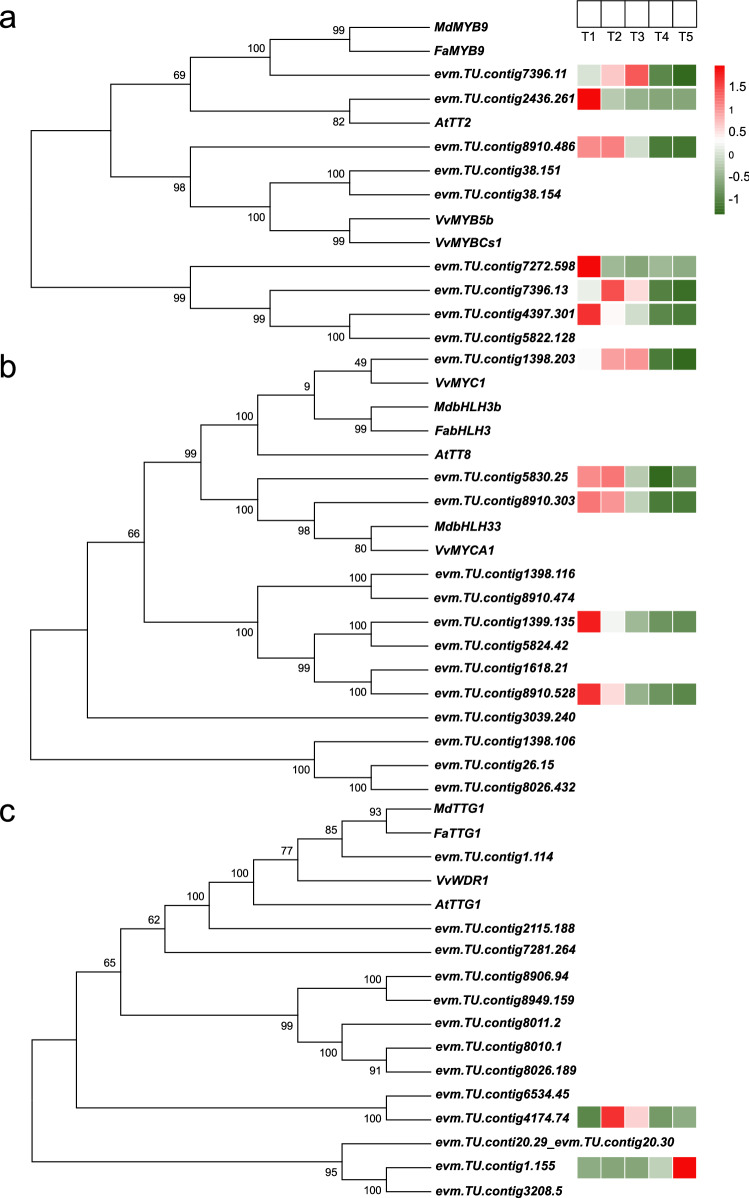
Phylogenetic trees of the *MYB*-*bHLH*-*WD40* complex members in C-PCNA persimmon. (**a**) Phylogenetic tree of *R2R3MYB*, (**b**) phylogenetic tree of *bHLH*, and (**c**) phylogenetic tree of *WD40*. Heat maps depict the normalized gene expression values, which represent the mean value of three biological replicates.

It has been reported that an MBW complex consisting of *MYB*, *bHLH*, and *WD40* is a critical factor for PA biosynthesis. Our study discovered a total of 14 *R2R3-MYB*, 13 *bHLH*, and 13 *WD40* transcription factors from the *D.kaki* genome. These transcription factors clustered with homologous *MYB*-*bHLH*-*WD40* complex genes of *A. thaliana*, *V. vinifera,* and *F. ananassa* (Fig. 5a–c). Among these transcription factors, only 6 *R2R3-MYB*, 5 *bHLH*, and 2 *WD40* genes were differentially expressed during C-PCNA persimmon fruit development, so these were used for further analysis. Of the 6 *R2R3-MYB* family genes, 5 showed significantly decreased expression at T4 vs T3. The *evm.TU.contig8910.486* and *evm.TU.contig7396.13* gene expression showed a remarkably positive correlation with total PA content (*P* < 0.05), where *evm.TU.contig8910.486* was from cluster 2. All 5 *bHLH* family genes were downregulated at T4 vs T3, and of these 5 genes, the expression of *evm.TU.contig1398.203* and *evm.TU.contig8910.303* cluster 2 genes presented a positive correlation with total PA content (*P* < 0.05). Only 1 *WD40* gene, *evm.TU.contig1.155,* from cluster 5 exhibited a significantly negative correlation with soluble PA content (*P* < 0.01).

### Weighted gene coexpression network analysis

**Figure 6:**
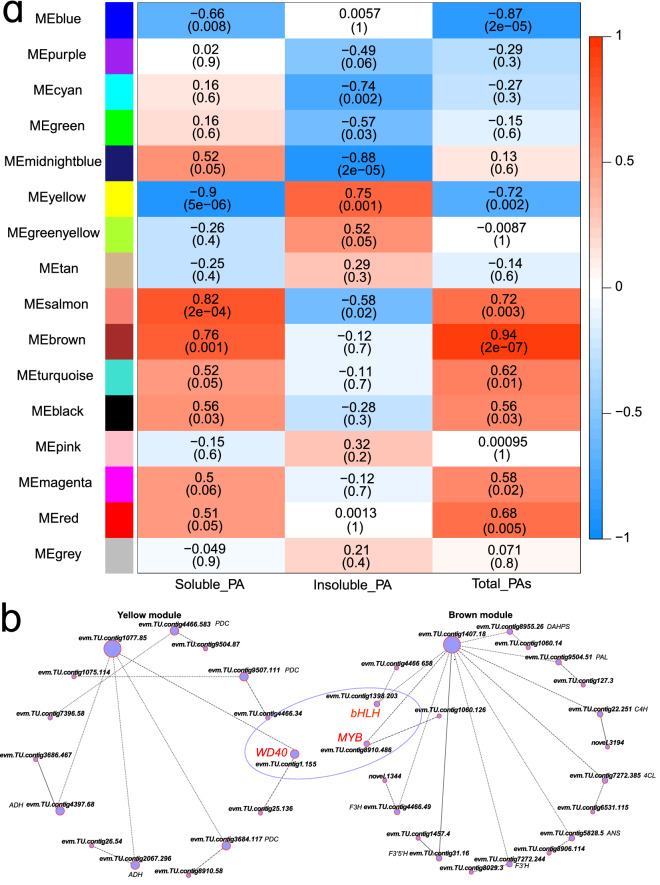
Weighted gene coexpression network analysis. (**a**) Gene modules defined using WGCNA and the association with soluble PA, insoluble PA, and total PA content. The numbers in the heatmap show the *P*-value (lower) and the correlation (upper). (**b**) The linkages of PA biosynthesis and insolubilization DEGs in yellow and brown modules, and the circles are sized by the gene connectivity.

To construct a potential regulatory network of PA accumulation in the C-PCNA persimmon, weighted gene coexpression network analysis (WGCNA) was performed using 7,102 DEGs, and 15 merged coexpression gene modules were identified. Among these modules, the brown module contained 904 genes, which had a significant and positive correlation with soluble PA (r = 0.76, *P* = 0.001) and total PA content (r = 0.94, *P* = 2e^−07^). The yellow module (864 DEGs) had a significant negative correlation with insoluble PA content (r = 0.75, *P* = 0.001) (Fig. 6a). This indicates that the brown and yellow modules may have a closer association and more significant correlations with PA biosynthesis and PA insolubilization, respectively.

Strikingly, the brown module genes included the structural genes *DAHPS* (*evm.TU.contig8955.26*), *PAL* (*evm.TU.contig9504.51*), *C4H* (*evm.TU.contig22.251*), *4CL* (*evm.TU.contig7272.385*), *ANS* (*evm.TU.contig5828.5*), *F3'H* (*evm.TU.contig7272.244*), *F3′5'H* (*evm.TU.contig31.16*), and *F3H* (*evm.TU.contig4466.49*), which have been linked to PA biosynthesis. Yellow module genes included the structural genes *PDC* (*evm.TU.contig4466.583*, *evm.TU.contig9507.111*, *evm.TU.contig3684.117*) and *ADH* (*evm.TU.contig4397.68*, *evm.TU.contig2067.296*), which have been linked to PA insolubilization. The *evm.TU.contig1407.18* (probable aminotransferase *ACS10*) and *evm.TU.contig1077.85* (*AHA10*) had high interconnections in the brown module and the yellow module, respectively. In addition, the critical transcription factors *MYB* (*evm.TU.contig8910.486*), *bHLH* (*evm.TU.contig1398.203*), and *WD40* (*evm.TU.contig1.155*) were identified. Among them, the *WD40* transcription factor in the yellow module was connected with the PA insolubilization genes *ADH* and *PDC* and presented a significant negative correlation with soluble PA content. Combined with the *MYB*-*bHLH*-*WD40* complex analysis above, *WD40* probably interacts with *MYB* (*evm.TU.contig8910.486*) and *bHLH* (*evm.TU.contig1398.203*) in the brown module, connecting and coregulating PA biosynthesis and insolubilization in C-PCNA persimmon (Fig. 6b).

## Discussion

An overview of genes and transcription factors associated with PA content variation in C-PCNA persimmon fruits at five late stages was obtained using the transcriptome based on a well-annotated C-PCNA persimmon genome. According to previous studies, research on the natural deastringency of the C-PCNA genotype has always been more inclined towards fruit that underwent 40 °C water deastringent treatment^21^ or the whole development stages as materials^38^, rather than using fruit at late developmental stages, which is more critical for natural C-PCNA deastringency. In the present study, we analyzed PA content and constructed C-PCNA genotype libraries representing five late development stages and used the transcriptome to evaluate differentially expressed genes and TFs associated with PA variation to identify potential PA biosynthesis-related and insolubilization-related genes.

Comprehensive analysis of phenotype (Fig. 1a), PA content (Fig. 1b), and DEG enrichment (Fig. S2) at five late developmental stages of the C-PCNA persimmon ‘Xiaoguo-tianshi’ helped us pinpoint the critical phase for PA variation. The C-PCNA persimmon fruit experiences the fastest growth stage from 120 to 140 DAF, where the soluble, insoluble, and total PA content was decreased. Moreover, the enrichment of “flavonoid biosynthesis” at this time point was related to PA biosynthesis^39^. These results indicated that 120–140 DAF could be the critical phase for the “dilution effect”. Soluble PA decreased while insoluble PA levels for C-PCNA persimmon increased rapidly from 140 to 160 DAF. Meanwhile, GO terms such as “drug transmembrane transporter activity” relating to PA transport^40^ and KEGG term “flavonoid biosynthesis” were significantly enriched. The results showed that 140–160 DAF could be the critical phase for PA transport and the “coagulation effect”. These results are consistent with previous research showing that C-PCNA persimmon undergoes deastringency at the late stage^6^.

Chorismate acid links primary and secondary metabolism in the shikimate pathway, which refers to the synthesis of phenylalanine from erythrose 4-phosphate and phosphoenolpyruvate in several steps^41^. It has been reported previously that inhibiting the *DAHPS* or *DHD/SDH* of the shikimate pathway results in the reduction of some secondary metabolites, such as chlorogenate and lignin, in tobacco and potato plants^42,43^. In our study, it was revealed that 1 *DAHPS* and 1 *DHD/SDH* were aggregated in cluster 2 and were downregulated between T4 vs T3 and T5 vs T4, which matched the proanthocyanin content. Downregulation of *DHD/SDH* has been reported in J-PCNA^9^, C-PCNA^5^, and artificially treated PCA persimmon^44^. However, *DAHPS* downregulation was observed in C-PCNA and C-PCNA persimmon that underwent artificial 40 °C water treatment^21^. These results showed that termination of PA accumulation accompanies downregulation of *DAHPS* and *DHD/SDH* in persimmon fruit.

Biosynthesis of the PA precursor flavan-3-ols shares the phenylpropane metabolic pathway, flavonoid synthesis and proanthocyanidin pathway^9,45^. *LAR* and *ANR* encode key enzymes involved in PA biosynthesis, which act in the production of 2,3-trans-flavan-3-ols [(+)-catechin (CA), and (+)-gallocatechin (GC)], 2,3-cis-flavan-3-ols [(−)-epicatechin (EC), (−)-epigallocatechin (ECG), and (−)-epi-gallocatechin 3-O-gallate (EGCG)], respectively^44^. We found that the expression of the phenylpropane and flavonoid structural genes *PAL*, *C4H*, *4CL*, *ANR*, *ANS*, *CHS*, *F3’H*, *F3′5'H,* and *F3H* decreased at T4 compared to T3, which was consistent with the decreased PA level. However, the *LAR* gene showed no differential expression in any developmental stage, which caused a reduction in epigallocatechin (EGC) and EGCG. This was one of the main reasons for PA reduction in the PCNA genotype^9^. Moreover, overexpression of Chinese Bayberry *MrANR* and *MrLAR* in tobacco indicated that *MrANR* increased PA accumulation, while *MrLAR* was unable to affect the total PA contents^46^. These results revealed that *ANR* was highly downregulated at the late stage of persimmon, which might be associated with the decrease in PA content by inhibiting PA precursor biosynthesis.

Previous studies suggest that acetaldehyde metabolism plays an important role in the astringency loss in both C-PCNA genotype^21^ and PCA persimmon with artificial deastringency treatment^47^. In plants, pyruvate is converted to acetaldehyde by *PDC*, then to ethanol by *ADH,* and acetate by mitochondrial *ALDH2a* and *ALDH2b* during the acetaldehyde biosynthesis process^48,49^. We found that 3 *ADH* and 2 *ALDH* genes that grouped in cluster 2 were downregulated, and 2 *ADH* and 1 *ALDH* that grouped in cluster 5 were upregulated. Three *PDC* genes were found to aggregate in cluster 5 and were upregulated between T5 and T4, which matched the insoluble PA content. The *ADH-like* and *ALDH2* genes were downregulated in the C-PCNA genotype natural deastringency process, and *PDC2* was specifically upregulated^21^. However, the expression patterns of the *DkADH1,3* and *DkPDC1,2* genes were upregulated in PCA persimmon during artificial ethylene deastringency treatment^47^, and 1 *ADH-like* gene showed high expression in 40 °C water treatment^21^. The upregulation of *ADH* and *PDC* genes to ethylene was also reported in other fruits, such as apricot^50^ and melon^51^, implying that the decrease in *ALDH* genes and the increase in *ADH* and *PDC* genes might result in acetaldehyde accumulation, causing C-PCNA deastringency naturally via PA insolubilization.

PA and anthocyanin biosynthesis pathway genes are mainly regulated by the MBW complex in plants, such as *Medicago truncatula*^52^ and *Anthurium andraeanum*^53^. *MY*B transcription factors are core members of the MBW complex. *MYB182* overexpression results in the reduction of PA and anthocyanin levels in poplar by reducing the expression of flavonoid biosynthesis structural genes^54^. *DkMYB14* in persimmon acts as both a repressor in PA biosynthesis and an activator in acetaldehyde biosynthesis^19^. *MYB* and *bHLH* autonomously mediate the expression of genes involved in the middle steps of the phenylpropanoid pathway^16^. In the ‘Red Delicious’, only a few *MYB* and *bHLH* members in the fruit skin were significantly correlated with anthocyanin content and suggested to regulate anthocyanin accumulation^55^. *WD40* proteins in the MBW complex are thought to confer a docking platform for the *MYB*–*bHLH* interaction^56^. A member of the MBW protein complex has been identified in persimmon through homologous cloning^20^ and transcriptome sequencing^21^. *DkMYB2*, *DkMYB4,* and *DkMYC1* (MBW) cooperatively increase the expression of a persimmon *ANR* gene involved in the biosynthesis of PA precursor cis-flavan-3-ols, which not only echoes the above analysis of PA biosynthesis structural genes, but also supports the presence of MBW complexes in persimmon^57^. We also identified *R2R3MYB*, *bHLH*, and *WD40* members based on the unpublished C-PCNA persimmon genome and indicated that the expression of the transcription factors *R2R3-MYB* (*evm.TU.contig8910.486* and *evm.TU.contig7396.13*) and *bHLH* (*evm.TU.contig1398.203* and *evm.TU.contig8910.303*) positively correlated with the total PA content and *WD40* (*evm.TU.contig1.155*) negatively correlated with the soluble PA content. These data provide the basis for targeted gene resources of transcription factors involved in PA accumulation in C-PCNA persimmon.

To reveal the potential regulatory network underlying PA content variation in C-PCNA persimmon, we performed WGCNA to identify modules of highly correlated genes associated with soluble PA, insoluble PA, and total PA content. We found that most PA biosynthesis and insolubilization DEGs were included in the brown and yellow modules, which were highly correlated with the soluble, insoluble, and total PA contents of the C-PCNA persimmon. A network of genes that were highly coexpressed with these PA biosynthesis-related and insolubilization-related DEGs was constructed. Among these genes, an MBW complex homologous *WD40* gene (*evm.TU.contig1.155*) showed a high correlation with the PA insolubilization genes *ADH* and *PDC* in WGCNA. This *WD40* gene also presented a highly negative correlation with soluble PA content. Previous studies have reported that the expression pattern of *DkWD40* was consistent with that of PA insolubilization genes such as *ADH*, and it is also consistent with the transformation of soluble PA to insoluble PA in the “Nishimura-wase” persimmon^58^. Combining WGCNA and phylogenetic analysis of the MBW complex, we found that the *WD40* gene (*evm.TU.contig1.155*), *MYB* (*evm.TU.contig8910.486*) and *bHLH* (*evm.TU.contig1398.203*) might be linked between PA biosynthesis and PA insolubilization. In this study, *evm.TU.contig1407.18* (probable aminotransferase *ACS10*) and *evm.TU.contig1077.85* (*AHA10*) were coexpressed with the *WD40* gene (*evm.TU.contig1.155*), *MYB* (*evm.TU.contig8910.486*) and *bHLH* (*evm.TU.contig1398.203*) and harbored the highest connections in the brown and yellow modules, respectively. *ACS* is the key enzyme in the ethylene biosynthetic pathway in plants^59^. In Arabidopsis, *AtACS10* is not ACC synthase but aminotransferase, it was clustered with *alanine AT* and *aspartate AT*, which speculated that it might encode aminotransferase^60^. Complementation experiments showed that *AtACS10* and *AtACS12* were ATases with broad specificity for aspartic acid and aromatic amino acids such as tyrosine and phenylalanine^60^. Besides, *aspartate AT*, the homologous genes of *AtACS10*, catalyze the synthesis of compounds such as phenylalanine via a transamination reaction^61^. Phenylalanine is the precursor of many flavonoid compounds such as PA, anthocyanins, and isoflavonoids^62^. The conversion of phenylalanine to PA and anthocyanins was catalyzed by enzymes such as *PAL*, *C4H*, and *4CL*^63^, which is consistent with our results that *ACS10* and the genes encoding these enzymes were coexpressed. *AHA10* that have been associated with PA accumulation with the disturbance of vacuolar acidification activates *TT12* to transport PA into vacuoles^64^. Thus, *evm.TU.contig1407.18* (probable aminotransferase *ACS10*) and *evm.TU.contig1077.85* (*AHA10*) might play important roles in the regulation of PA accumulation. The overall results suggest that the *MBW* complex might play important roles in regulating PA biosynthesis and PA insolubilization; however, the mechanism remains unclear and requires further investigation.

## Conclusion

Transcriptome data of C-PCNA persimmon fruit at five developmental stages have provided the specific processes that lead to PA biosynthesis and PA insolubilization. Altogether, the physiological, PA content, and transcriptome data revealed that 120 to 140 DAF is the critical phase for PA biosynthesis, while 140 to 160 DAF is the critical phase for PA transport and PA insolubilization. The results indicate that the downregulation of the *ANR* gene at T5 vs T4 may be associated with a reduction in PA biosynthesis by inhibiting its precursor cis-flavan-3-ols. The decrease in *ALDH* and an increase in *ADH* and *PDC* genes might result in C-PCNA persimmon’s PA insolubilization. MBW complex homologous *R2R3-MYB* (*evm.TU.contig8910.486* and *evm.TU.contig7396.13*), bHLH (*evm.TU.contig1398.203* and *evm.TU.contig8910.303*), and *WD40* (*evm.TU.contig1.155*) were isolated from the C-PCNA *D.kaki* genome (unpublished) and were highly correlated with PA content. In addition, the *WD40* (*evm.TU.contig1.155*), *MYB* (*evm.TU.contig8910.486*) and *bHLH* (*evm.TU.contig1398.203*) genes might link and coregulate both PA biosynthesis and insolubilization via WGCNA. These genes might play important roles in PA content variation. This study laid an empirical foundation for ongoing investigations of PA biosynthesis and PA insolubilization in C-PCNA persimmon.

## Supplementary Information

Supplementary Information.

## Data Availability

The raw transcriptome sequencing data were deposited in the National Center for Biotechnology Information Sequence Read Archive (NCBI SRA) under Bioproject ID PRJNA771936.
