# Supplementary material for: Transcriptomic profiling analysis to identify genes associated with PA biosynthesis and insolubilization in the late stage of fruit development in C-PCNA persimmon
Source: Sci Rep. 2022 Nov 9;12:19140. doi: 10.1038/s41598-022-23742-4 (PMC9646812; doi:10.1038/s41598-022-23742-4)
Supplement: Supplementary file 1 — Supplementary Information. [file 41598_2022_23742_MOESM1_ESM.docx]

Supplementary Information

**Transcriptomic Profiling Analysis to Identify Genes Associated with PA biosynthesis and Insolubilization in the Late Stage of Fruit Development in C-PCNA Persimmon**

Yiru Wang^1, 3^, Qi Zhang^1, 2, 3^, Tingting Pu^1, 2^, Yujing Suo^1, *^, Weijuan Han^1^, Songfeng Diao^1^, Huawei Li^1^, Peng Sun^1^, Jianmin Fu^1, *^

^1^Research Institute of Non-timber Forestry, Chinese Academy of Forestry, Key Laboratory of Non-timber Forest Germplasm Enhancement & Utilization of State Administration of Forestry and Grassland, No. 3 Weiwu Road, Jinshui District, Zhengzhou 450003, China.

^2^College of Forestry, Inner Mongolia Agricultural University, Hohhot 010018, China.

^3^These authors contributed equally: Yiru Wang and Qi Zhang.

⁎ Corresponding author

E-mail addresses: suoyujing1988@126.com (Y. S.), fjm371@163.com (J. F.).


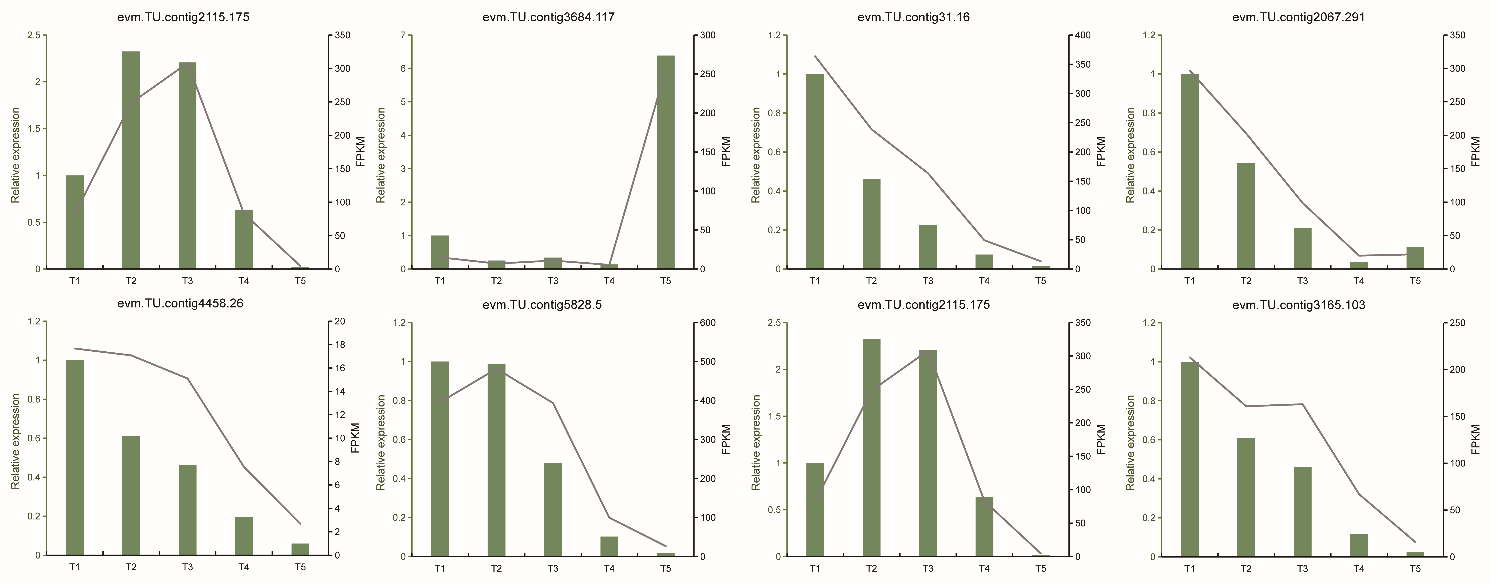


**Supplementary Figure S1.** Verification of transcriptomic data by qRT-PCR analysis of 8 gene expression.


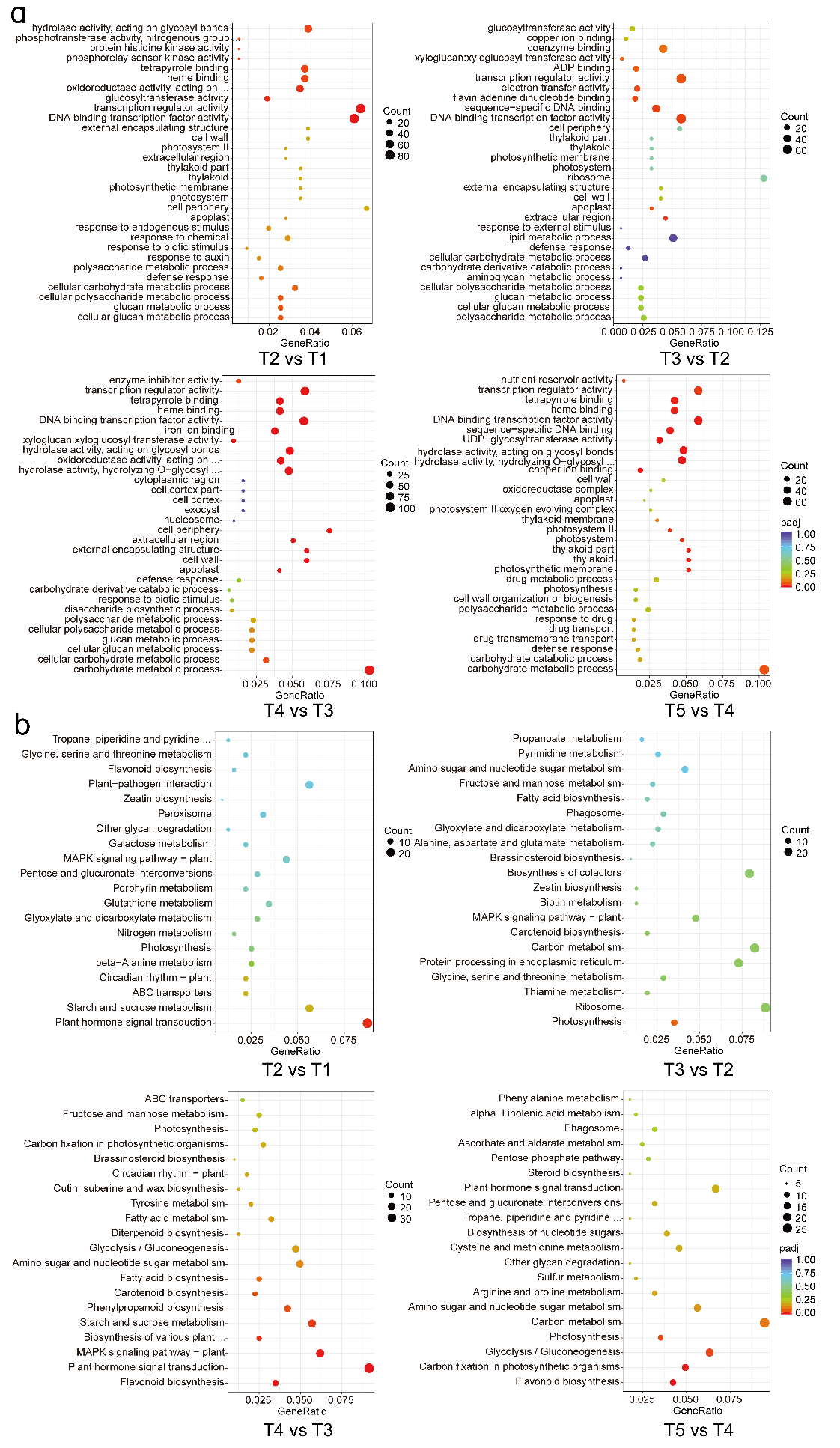


**Supplementary Figure S2.** The GO **(a)** and KEGG **(b)** analysis of DEGs in four comparison groups (T2 vs T1, T3 vs T2, T4 vs T3 and T5 vs T4).

| **Supplementary table S1** The primers for RT-qPCR | | | |  |  |
| --- | --- | --- | --- | --- | --- |
| ID | | Forward Primer (5' to 3') | Reverse Primer (5' to 3') |  |  |
| evm.TU.contig3165.103 | | TCATAGGCAAGCATGGTGTTTCCC | GGGCTGCATTATCAGTCTCCTTCAG |  |  |
| evm.TU.contig2115.175 | | TCCGTCAAGCGCCTCATGAT | TCGGAGCAGACAACGAGGAC |  |  |
| evm.TU.contig4466.754 | | TGCCCATGTGGAGGATGTTTGC | CTGGAGCACTGGTATTGACAGCAC |  |  |
| evm.TU.contig2067.291 | | GGGACCTTCTTCGGCAACTACAAG | GTTGATCTCCGAGAATGGCACCTC |  |  |
| evm.TU.contig5828.5 | | AGGATGAGAAGGCGAGGCAGAG | CGGCAGGTTGAAGAAGGCATCG |  |  |
| evm.TU.contig4458.26 | | CTCAGGCGATGGCTTGGAATACC | CGCTTGTTTACCTGTTTGCTTACCG |  |  |
| evm.TU.contig31.16 | | CAACGTGGTTGTGGCGTCAA | GCATCGTAGGCCAGATGGGT |  |  |
| evm.TU.contig3684.117 | | GCTACTCGCTGCTGCTCAAGAAG | TTCGCCAGAGCCTTCAAGAAATCC |  |  |
| **Supplementary table S2.** The sequence of MYB-bHLH-WD40 | | | | | |
| *ID* | | Sequence | | | |
| *AtTT2* | | MGKRATTSVRREELNRGAWTDHEDKILRDYITTHGEGKWSTLPNQAGLKRCGKSCRLRWKNYLRPGIKRGNISSDEEELIIRLHNLLGNRWSLIAGRLPGRTDNEIKNHWNSNLRKRLPKTQTKQPKRIKHSTNNENNVCVIRTKAIRCSKTLLFSDLSLQKKSSTSPLPLKEQEMDQGGSSLMGDLEFDFDRIHSEFHFPDLMDFDGLDCGNVTSLVSSNEILGELVPAQGNLDLNRPFTSCHHRGDDEDWLRDFTC | | | |
| *MdMYB11* | | MGRSPCCSKDEGLNRGAWTAMEDKVLTEYIGNHGEGKWRNLPKRAGLKRCGKSCRLRWLNYLRPDIKRGNITRDEEELIIRLHKLLGNRWSLIAGRLPGRTDNEIKNYWNTTIGKRIQVEGRSCSDGNRRPTQEKPKPTLSPKPSTNISCTKVVRTKASRCTKVVLPHESQKFGYSTEQVVNAAPTLNQAVNNPMVGIDEPLLPMSFLDDENNNSCEFLVDFKMDENFLSDFLNVDFSVLYNNEGAGKAAAAATTEDTSNKLHGPDLRSSKAPIIESELDCWLAFDRRSSAMATTESPGAVKKVVVHLRATGDAPILKQSKFKILGTDKFAKVIEFLRRQLHKETLVNTKEFILQKQREREFSSIETSEQVEIR* | | | |
| *MdMYB9* | | MGRSPCCSKEGLNRGAWTALEDKILSSYIKAHGEGKWRSLPKRAGLKRCGKSCRLRWLNYLRPDIKRGNISGDEEELIVRLHNLLGNRWSLIAGRLPGRTDNEIKNYWNTTLGKKSKVDSFSGSSKETSLNPCKSIAKKKDVESKTSTAAAQPLVIRTKATRLTKILVPQNIPSDENYTAAAANPLELQTQSAEKGGSTEEFPRTNAGDCSNILKNFGCDDDDIDAKGDQYCNEFQLLNSIPLDEAXINDGCWTGGNGCDLEDYGASLDLDSLAFLLDSEEWPSQENVVV | | | |
| *VvMYB5b* | | MRNASSASAPPSSSSKTPCCIKVGLKRGPWTPEEDEVLANYIKKEGEGRWRTLPKRAGLLRCGKSCRLRWMNYLRPSVKRGQIAPDEEDLILRLHRLLGNRWALIAGRIPGRTDNEIKNYWNTHLSKKLISQGIDPRTHKPLNPNSSSVDVKASSSKAKAVMNPNPNPNPSPSEKAAANKEAGNFKSDNQYQIGAAGNDGSANIQNSDGSGTGLRSSNNEEDDDLNCGTDDVFSSFLNSLINEDVFPGQHHLQQQHHGGLIAPGSDALISTSSVQSFGFGTSWEAAAMTSTSVFSQIDHSKRFNDQPDKRF | | | |
| *VvMYBCs1* | | MRNPASASTSKTPCCTKVGLKRGPWTPEEDELLANYVKREGEGRWRTLPKRAGLLRCGKSCRLRWMNYLRPSVKRGQIAPDEEDLILRLHRLLGNRWSLIAGRIPGRTDNEIKNYWNTHLSKKLISQGIDPRTHKPLNPKPNPSPDVNAPVSKSIPNANPNPSSSRVGEIGSNHEVKEIESNENHKEPPNLDQYHSPLAADSNENWQSADGLVTGLQSTHGTSNDDEDDIGFCNDDTFPSFLNSLINEDVFGNHNHHQQQQQQQQLQQVQQPSNVIAPLPHPAISVQATFSSSPRTVWEPAALTSTSAPLVHDQKHSMSP | | | |
| *FaMYB11* | | MGRSPCCAKEGLNRGAWTAMEDRTLTEYITTHGEGKWRNLPKRAGLKRCGKSCRLRWLNYLRPDIKRGNITRDEEELIIRLHKLLGNRWSLIAGRLPGRTDNEIKNYWNTNIRKKVQDHSSTNSEANITHHKPPNHQTQKKNTNVVRTKASRCTKVFMPHQQSQMDKKGTCNNPTADQQGAAPFLNHDYYDPINYNDDPALRMMGITDTHHQESDDLSPFLNLEIDNENSNSCGFMVDFKMDESFLSEFLNVDFSELYSSSTSTANGGDGVKAVINNSCGDNDHHELHSPDFGSSMAPIIDSEVDWLS | | | |
| *FaMYB9* | | MGRSPCCSKEGLNRGAWTALEDKVLTSYIKAHGEGKWRNLPKRAGLKRCGKSCRLRWLNYLRPDIKRGNISGDEEELIIRLHNLLGNRWSLIAGRLPGRTDNEIKNYWNTTLSKKAKPESHSGSSKETSPGPTRFRPRKASAAATTQPQVIRTKATRLTRMPVPSLPLLIDDCSTSTTALELQVPQTQLVSSLPEDAVNTQVHFQGTDAMNFGCNGFQATAGDDEDAKGDYDIPLDDGMLNDWTGNGNCDLENYGASLDLDSLAFLLDSDD | | | |
| *AtTT8* | | MDESSIIPAEKVAGAEKKELQGLLKTAVQSVDWTYSVFWQFCPQQRVLVWGNGYYNGAIKTRKTTQPAEVTAEEAALERSQQLRELYETLLAGESTSEARACTALSPEDLTETEWFYLMCVSFSFPPPSGMPGKAYARRKHVWLSGANEVDSKTFSRAILAKSAKIQTVVCIPMLDGVVELGTTKKVREDVEFVELTKSFFYDHCKTNPKPALSEHSTYEVHEEAEDEEEVEEEMTMSEEMRLGSPDDEDVSNQNLHSDLHIESTHTLDTHMDMMNLMEEGGNYSQTVTTLLMSHPTSLLSDSVSTSSYIQSSFATWRVENGKEHQQVKTAPSSQWVLKQMIFRVPFLHDNTKDKRLPREDLSHVVAERRRREKLNEKFITLRSMVPFVTKMDKVSILGDTIAYVNHLRKRVHELENTHHEQQHKRTRTCKRKTSEEVEVSIIENDVLLEMRCEYRDGLLLDILQVLHELGIETTAVHTSVNDHDFEAEIRAKVRGKKASIAEVKRAIHQVIIHDTNL | | | |
| *MdbHLH3b* | | MELLQLSSSRSCALEMPFGTHHMQLPGKAYARRQHVWLTGANEVDSKTFSRAILAKSARIQTVVCIPLLDGVVEFGTTERVPEDHAFVQRVKTFFVDHHHPPPPKPALSEHSTSNPTTSSDHPHFHSSNLLPAMSTDPPLNAAQEDDEDEEDDNQEEDDGAESDSEAETGPNGGAIVPAANPPQALAAVAEPSELMQLEMSEDIRLGSPDDASNNLDSDFHLLALSQSRNPADQQRQADSYRAESTRRWPSVQEPLSSGLQPPHSGPXALEELTNDDTHYSETVSTILQGQSARWMMNSSTDYAVCSTQSAFAKWSSRVDHHLLMPVEGTSQWLLKYILFSVPFLHSKYRDENSPKSHEGEASTRLRKGTPQDELSANHVLAERRRREKLNERFIILRSLVPFVTKMDKASILGDTIEYVKQLRKKIQDLEARNVPMEDDQQSRSSGEMQRSSSCKELRSGLPVVERTQAGHGSDKRKLRIVEGSGGVAIAKPKAMEDLPPPLPPPPPQPEPSPTPMVTGTSLEVSIIESDGLLELQCPYREGLLLDVMQTLRELRIETXVVHSSLNNGYFVAELRAKVKDNVNGKKISITEVKRVINQIIPQSDS* | | | |
| *MdbHLH33* | | MAQNHERVPGNLRKQFAVAVRSIKWSYAIFWSLSTTQQGVLEWGEGYYNGDIKTRKKVEGVELKTDKMGLQRNVQLRELYKSLLEGETETEQQAKAPSAVLSPEDLTDAEWYYLLCMSFIFNPGEGLPGRALATGQTIWLCNAQHTDSKVFSRSLLAKSASVQTVVCFPYLGGVVELGVTELVSEDLNLIQHIKASLLDFSKPDCCEKSSSAPHKPDDDSEQIVAKVDHDVVDTLPLENLYSPSEEIKFDQRGINGLLGNHEEVNMDSSDECSNGCDHNHPTEDSMMLEGTNAVASQVQSWHFMDEDFSSGVQDSMNSSDSISEAFVNQGKAHSFAKHENANHIHLKELQNFNDTKLSSLYLGSVDEHVHYKRTLCTLLGSSMKLIENPCFCDGESKSSFVKWKKEVVGSCRPTVHQKTLKKILFTVPLMYGVHSPMATGKENTGKDLLPNLQGDDINREHDKMRENAKLLVLRSMVPSITEVDKASILDDTIKYLKELEARAEEMESCMDTVEAISRGKFLNRVEKTSDNYDKTKKNNVKKSLVKKRKACDIDETDPYPNMLVSGESLPLDVKVCVKEQEVLIEMRCPYREYILLDIMDAINNLYLDAHSVQSSILDGVLTLSLKSKFRGAAISPVGMIKQVLWKIAGKC | | | |
| *VvMYC1* | | MAAPPNSRLQSMLQSAVQSVRWTYSLFWQICPQQGILVWGDGYYNGAIKTRKTVQPMEVSAEEASLQRSQQLRELYESLSAGETNQPARRPCAALSPEDLTESEWFYLMCVSFSFPPGVGLPGKAYAKRHHIWLAGANEVDSKVFSRAILAKSARVQTVVCIPLMDGVVEFGTTEKVQEDLGFVQHVKSFFTDHHLHNHPPKPALSEHSTSNPATSSDHSRFHSPPIQAAYAAADPPASNNQEEEEEEEEEEEEEEEEEEEEEEEEAESDSEAETGRNNRRVRTQNTGTEGVAGSHTAAEPSELIQLEMSEGIRLGSPDDGSNNLDSDFHMLAVSQPGSSVDHQRRADSYRAESARRWPMLQDPLCSSGLQQPPPQPPTGPPPLDELSQEDTHYSQTVSTILQHQPNRWSESSSSGCIAPYSSQSAFAKWTTRCDHHHHPMAVEGTSQWLLKYILFSVPFLHTKYRDENSPKSRDGDSAGRFRKGTPQDELSANHVLAERRRREKLNERFIILRSLVPFVTKMDKASILGDTIEYVKQLRKKIQDLEARTRQMEVEQRSRGSDSVRSKEHRIGSGSVDRNRAVVAGSDKRKLRIVEGSTGAKPKVVDSPPAAVEGGTTTVEVSIIESDALLEMQCPYREGLLLDVMQMLRELRLETTTVQSSLTNGVFVAELRAKVKENASGKKASIMEVKRAINQIIPQC | | | |
| *VvMYCA1* | | MANGVQNQEGVPENLSKQLAVAVRSIQWSYAIFWSLSTRQQGVLEWSGGYYNGDIKTRKTVQEMELKADKMGLQRSEQLRELYESLLEGETDQQSKRPSAALSPEDLSDAEWYYLVCMSFVFNPGEGLPGRALANGQSIWLCDAQYADSKVFSRSLLAKTVVCFPHMGGVIELGVTELVPEDPSLIQHIKACLLELSKPICSEKSSFVPCNTDDDKDRMCAKVDHDIVETMALEKLYPATEEIKFEQEGMSELHGNIHEEHNIGSPDDCSNGCEDDHQTEDSFMLEGINGGASQVQSWHFVDDDFSNGVQGSMDSSDCISQAFVNQERIHSSPKGENVNNVRLKDLQECNDTKFSSLDLGADDDLHYRRTISTVLRKSHPLIGNSCFRCYDIKSSFITWKKGGMLDAQKPQTQQRILKKILFTVPLMHGGCGFKSQKENAGRDGLWKSGSDGICKQHALSDKKREKEKFLVLRSMVPSINKIDEVSILGDTIEYLKKLEARVEELETSMDLQTELDARARQKYLDMVEQTSDNYDDKMIDDGKKLWINKRKACDIDETDLEINEIIPKDSLPSSDMKVRINEQEVLIEMRCPWREYLLLDIMDAINNLHLDCHSVQSSNHDGFLTLTLKSKFRGRAVASAGMIKQALWRITSKC | | | |
| *FabHLH3* | | MATPPPSSSRLRGMLQSAVQSVQWTYSLFWQICPQQGMLXXXMXYYNGAIKTRKTVQPMEVSSEEASLQRSQQLRELYDTLSAGETNQPARRPCAALSPEDLTESEWFYLMCVSFSFPPGVGLPGKAYSRRQHVWLTGANEVDSKTFSRAILAKSARVQTVVCIPLLDGVVELGTTDRVPEDLAFVQHVKTFFVDHHHLPPPKPALSEHSTSNPATSSDHHPRFHSPHLTAISNNAAANNPPPLHAAEQEDDENEDEEGESDSEAETGRNGNGSPLPPAGTNVPSAEPSELMQLEMSEDIRLGSPDDASNNLDSDFHMLAVSQSANAADQQRQADSYRAESARRWPPVQRPMSAVQPPPSGSLELEELNHDDTHYSETVSTILQTQATRWTDSSSNDYVTYSIQSAFAKWTNRADHHLLMPVEGTSQWLLKYILFSVPFLHTKYRDENSPKSSHDGEGSTRLRKGTSQDELSANHVLAERRRREKLNERFIILRSLVPFVTKMDKASILGDTIEYVKQLRKKIKDLEARNVHLEDDQQHTRSLGEIQRSSSMKELRSALTVTERSRVGSPGSDKRKLRIVEGSGGVAVAKPKVVENRHSSATTAPEPAPPMPMLTGTSLEVSIIESDGLLELHCPYREGLLLDVMQTLRDLRIETTVVQSSLNSGTFVAELRAKVGKKTTITEVKRAVNQVIPQSDS | | | |
| *AtTTG1* | | MDNSAPDSLSRSETAVTYDSPYPLYAMAFSSLRSSSGHRIAVGSFLEDYNNRIDILSFDSDSMTVKPLPNLSFEHPYPPTKLMFSPPSLRRPSSGDLLASSGDFLRLWEINEDSSTVEPISVLNNSKTSEFCAPLTSFDWNDVEPKRLGTCSIDTTCTIWDIEKSVVETQLIAHDKEVHDIAWGEARVFASVSADGSVRIFDLRDKEHSTIIYESPQPDTPLLRLAWNKQDLRYMATILMDSNKVVILDIRSPTMPVAELERHQASVNAIAWAPQSCKHICSGGDDTQALIWELPTVAGPNGIDPMSVYSAGSEINQLQWSSSQPDWIGIAFANKMQLLRV | | | |
| *MdTTG1* | | MENSTQESHLRAENSVTYESPYPLYAMAFASPQTRTRHQHHRIAVGSFIEEYSNRVDILSFDPDTLSIKPNPTLSFDHPYPPTKLMFHPNPNALHKTNDVLASSGDYLRLWEVGDSTVEPIQVLNNSKTSEFCAPLTSFDWNDIEPRRIGTSSIDTTCTIWDIEKGVVETQLIAHDKEVYDIAWGEARVFASVSADGSVRIFDLRDKEHSTIIYESPQPDTPLLRLAWNKQDLRYMATILMDSNKVVILDIRSPTMPVAELERHRGSVNAIAWAPQSCRHICSAGDDTQALIWDLPTVAGPNGIDPMSMYSAGAEINQLQWSAAQPDWISIAFSNKMQLLKV | | | |
| *VvWDR1* | | MERSSQESHLRSENCVTYESPYPLYSMAISSSTQHHHPRIAIGSFIEELSNRVDIVSFDEDALAIRTHPSLSFDHPYPPTKLMFHPNSRTSPDHLASSGEYLRLWEVRDNSIQPLSVLNNSKTSEFCAPLTSFDWNEVEPRRIGTSSIDTTCTIWDVERGVVETQLIAHDKEVYDIAWGEAGVFASVSADGSVRIFDLRDKEHSTIIYESPQPDTPLLRLAWNKQDVRYMATILMDSNKIVILDIRSPTMPVAELERHRASVNAISWAPQSSRHICSGGDDSQALIWELPTLAGPNGIDPMSVYLASSEINQLQWSAAQPEWIAIAFSNKLQLLKV | | | |
| *FaTTG1* | | MENSTLESHLGPENSVTYDSPYPLYAMAFSPAARTRHHHPKIAVGSFIEEFSNRVDILSFDPDTLTLKPNPSLSFDHPYPPTKLMFHPNPNTLHKSHDILASSGDYLRLWEVKDSSVDRLEPISVLNNSKTSEFCAPXTSFDWNEIEPRXIGTSSIDTTCTIWDIEKGVVETQLIAHDKEVYDIAWGEARVFASVSADGSVRIFDLRDKEHSTIIYESPQPDTPLLRLAWNKQDLRYMATILMDSNKVVILDIRSPTMPVAELERHRGSVNAIAWAPQSARHICSGGDDSQALIWELPTVAGPNGIDPMSMYSAGAEINQLQWSAGQPDWIAIAFSNKMQLLKV | | | |
| *evm.TU.contig4397.301* | | MKGSYGDHLSRVRGAKKGGCHLQGEGVYIKGAWTAEEDRKLAECIEVHGAKKWKTIAIKSGLNRCGKSCRLRWLNYLRPNIKRGNISHEEEDLILRLHKLLGNRWSLIAGRLPGRTDNEIKNYWNSHLGKKINQQGKAMATPAAQESPPQAAASAAEEEEEEHDGGATGGSRDHPENVMSFESVGGFFNFSAGDGTYGMEWVNKFLELDDDVDRWFAE* | | | |
| *evm.TU.contig38.151* | | MRQPPARSSAAAVKGATAATTVTAATTTTACCSKVGLKRGPWTPEEDELLSNYIKKEGEGRWRTLPKRAGLLRCGKSCRLRWMNYLRPSVKRGHISPDEEDLILRLHRLLGNRWSLIAGRIPGRTDNEIKNYWNTHLSKKLISQGIDPRTHKPLTNAATTTTTTASINHHHENPSSSSKPNNRVIMENPNPNNPAVATAAVSEEPATAGSGGVPLLAMRSAPTSGDDGTTGGGVTNLQIINSDHQVSAGMIMGLNIGNASCSSNQQDDDDEDDTINYCTDDVLSSFLNSLINEDLFENQNHAQIQHHTTVPPSAAAAVSFDPLISTTHQSVAFGAGWDAPIMFSSSDLNQTRDPKRLN* | | | |
| *evm.TU.contig38.154* | | MRQPAARLKGATAATTTTACCSKVGLKRGPWTPEEDELLSNYIKKEGEGRWRTLPKRAGLLRCGKSCRLRWMNYLRPSVKRGHISPDEEDLILRLHRLLGNRWSLIAGRIPGRTDNEIKNYWNTHLSKKLISQGIDPRTHKPLTNTSTSTSINHHHHHENPSSSSKPNNRIIMEEPATAGSGGVPLLAMRRAPATGDDGTTGGGVTNFQIINSDHQVSGGMMMGLNIGNASCSSNQQDDDDDDDDNDDSDTINYCTDDVLSSFLNSLMNEDLFENQHHAQIPHHTTVPPAAAAAASFDPLISTTHQSVAFGAGWDAPIMFSSSDLNQTPDPKRLN* | | | |
| *evm.TU.contig8910.486* | | MGRAPCCSKVGLHRGPWTGKEDALLTKYIQVHGEGSWRSLPKKAGLLRCGKSCRLRWMNYLRPDIKRGNITPDEDDLIIKMHALLGNRWSLIAGRLPGRTDNEIKNYWNTHLSKRLRSQGTDPNTHKKLSDSHVQEPKKRSSNKKQKNKSKSNLDHTEKLKVHNPKPFRIKSLASFSFSRDSSSFDWTTTTTTTTTTTATATPSGSSNHEGERGMLGNNGSNGHEVGFFIGEDGSDHYHHDHMMDGSDLECQSLEKLYEEYLQLLKTEEDDQGQLDSFAESLLI* | | | |
| *evm.TU.contig7272.598* | | MEELGKKAESARIKKGLWKPEEDMILKSYVEAYGEGKWATISQQSGLLRSGKSCRLRWKNYLRPNIKRGQMSDEEKDLIIRLHKLLGNRWSLIAGRLPGRTDNEVKNYWNTHLSKQHNYYSQRKTAAALTRAKNKKAQLRPPPTASTTPPICAEASVNGSKEEQITAITAPLMEETENLFTFDDIQPPSLSSATYNNAPFFFDDDELFMPILDSFALSEAFGSFVDEANYSHDHEHVG* | | | |
| *evm.TU.contig7396.13* | | MAPKKNEAVEIKPLNRGAWTAEEDRKLAQVIEIHGAKRWKTIAAMAGLNRSGKSCRLRWLNYLRPNIKRGNITDQEEDLILRLHKLLGNRWSLIAGRLPGRTDNEIKNYWNSRLSRKISQREKRGGAEDPSARESSGSELIRPPDQAAGGATMVDGDDDFFDFSNESPMTLEWVSKFLELEEDSYELS* | | | |
| *evm.TU.contig7396.11* | | MGRSPCCSKEGLNRGAWTAMEDKILTDYIKANGEGKWRNLPKRAGLKRCGKSCRLRWLNYLRPDIKRGNITHDEEDLIIRLHRLLGNRWSLIAGRLPGRTDNEIKNYWNTNIGKKVQGHPSAMSNRKPKGKPVAGKELRGHAVPNAAMESCVVRTKASRCTKVFITDPDPQHGASTFPGDHKPGPVAGSEPANEAPAGPANAMQDNGGSGCDQFMMMNFEVDDKFLSDFLNTDFAPLSEFDQTGGLELDGNINDTNTSSSSPENSDQTLLLSDDKLFNDSDFQFLDPASMDWLQEQQKIRGLNENNN* | | | |
| *evm.TU.contig5822.128* | | MAPMKGEGAYSKGAWTAEEDRKLAGCIEVHGAKKWKTVAIKSGLNRCGKSCRLRWLNYLRPNIKRGNISHEEEDLILRLHKLLGNRWSLIAGRLPGRTDNEIKNYWNSHLSKKINQQGKAMATPAAQESPPQAADCAAEEERHGGGATGSRDPENMSFESGEFLNFSGDGTYGLEWVNKFLELDDEDVDQWLAE* | | | |
| *evm.TU.contig2436.261* | | MPNQARLSWEQHPKREREREREGGREINIIMGRKPCCAKVGLNKGAWTAREDKILANYIKVHGEGKWRDMPQKAGLKRCGKSCRLRWLNYLRPDIKRGNISEEEEELILRLHKLLGNRWSLIARRLPGRTDNEIKNYWNSNLSKRINHGDNNYSKNSRKQRSPRPQKHTVIRTKAVRCTKVVIPQELNVTAMIEKCEAPAASQSDYLLDFDASGDLFVTDLLDLEIPLEPNENDGGGGDGGDFSGDVCGFPPSEAWWSLNDLSDEIILEADENWRASTEPPQTHGLG* | | | |
| *evm.TU.contig20.29_evm.TU.contig20.30* | | MMYGDPQQQQHHHQPPPMQPPQQQQHIPQVGEFPRGPPLPPPPMMRQSSASSTNMGGPPDFHQQHHHPPAPHPPYDVHGDSYGAKRIRKIGQRRAVDYTSTVVRYMQIRMWQRDSRDRTVLQPTPAAAIDMLPTAAYSDNPSTSFAAKFVHTSLNKNRCSINRVLWTPSGRRLITGSQSGEFTLWNGQSFNFEMILQAHDQAIRSMVWSYNDNWMVTGDDGGSIKYWQNNMNNVKANKSAHKESVRDLSFCRTDLKFCSCSDDTTVKVWDFARCQEERSLSGHGWDVKSVDWHPTKSLLVSGGKDNLVKLWDAKSGRELSSFHGHKNTVLCVKWNQNGNWVLTASKDQIIKLYDIRAMKELESFRGHRKDVTALAWHPFHEEYFVSGSFDGSIFHWLVGHETPQVEISSAHDNSVWDLAWHPIGYILCSGSNDHTTKFWCRNRPGDPARDKFNVGHSQGYGEQNPALAGRMPGNFQGPELPTTPGPFAAGLTRNEGTIPGVGVAMPLSISSLDTSTQGEQKPPMSMPLGAPPLPPGPHPSLLAANQQQAYQQNVQQAQQHHQPHPQRMTSLPLPPPNLPQLQPPSHLPILTHPHLARPPPHLPPLGMPSSMPSSMPMPGQMGMQGAMNQMVPPLQQGHFMMNPVHSGSAPPGGGIPSGLANMQAPTNASGTQMYQPGGAFNRPQAGPVPPMPGLNPYQPGNPNAGGMGGLPSNFGLQSGMPPPLPPGPPPQGKSTQ* | | | |
| *evm.TU.contig8906.94* | | MGKDEDEMRGEIEERLINEEYKIWKKNTPFLYDLVITHALEWPSLTVEWLPDREEPPGKDYSVQKMILGTHTSENEPNYLMLAQVQLPLEDAENDARHYDDDRSDFGGFGCANGKVQIIQQINHDGEVNRARYMPQNPFIIATKTVSAEVYVFDYSKHPSKPPLDGACNPDLRLRGHSTEGYGLSWSQFKQGHLLSGSDDAQICLWDINATPKNKALDAMQIFKVHEGVVEDVAWHLRHEYLFGSVGDDQYLHIWDLRTPSVSKPIQSVIAHQSEVNCLAFNPFNEWVVATGSTDKTVKLFDLRKISTSLHTFDCHKEEVFQVGWNPKNETILASCCLGRRLMVWDLSRIDEEQTPEDAEDGPPELLFIHGGHTSKISDFSWNPCEDWVIASVAEDNILQIWQMAENIYHDEDDIPADEPAKAS* | | | |
| *evm.TU.contig6534.45* | | MEGSSESGGQKSGVCEDEVPADTAVQAIEWSDVSLRQWLDNPERAVDAIECLHIFTQIVNIVNLAHSQGIVVHNVRPSCFVMSSFNHVSFIESASCSDSGSDSLEDGLNSQTAEFKELSSQVQDHSRQLTSQFGTENSQPETNQTNSSYVISETSCLQSSFVYPKQYSLEACSNVEQPEEKQHLFPMKQILLMETNWYSSPEEVAGDPSSCASDIYRLGVLLFELFCTFSSGEEKSSAMSSLRHRILPPQLLLKWPKEASFCLWLLHPDPSSRPKISDLLQSEFLNEPRENIEEREAAIDIREKIEEQELLLEFLLLIQQRKLEAANNLQDIISFLSSDIEEVTHQQSIIWRRGALCPEPSKDAESGLLMMGVVENNDYSCSKSRKRFRPGLRSHDPGESGNLLHEDHNVEGPAENEGSILSKGSRLSKNFKRLESAYFSARHGHIKPTGKSLTRRSPLSSDGGGSIIVTERNSVNNLPVRGQYNEARQSGWINSFLEGLCKYLSFSKLKVRADLKQGDLLKSPNLICSLSFDRDGEFFATAGVNKKIKVFEYDPIVNEHRDIHYPVVELASRSKLSCICWNSYIKSQIASSNFEGVVQVWDVTRGQVFTEMREHEKRVWSVDFSLADPTMLASGSDDGSVKLWNINQGVSVGTIRTKANVCCVQFPVDSGHSLAFGSADHRVYYYDLRNSRMPLCTLIGHNKTVSYVKFIDSTNLVSASTDNTLKLWDLSMCTSQVLDSPLQSFTGHRNVKNFVGLSVSEGYIATGSETNEVFIYHKAFPMPALSFKFSSVDPVSGDELDDPAQFISSVSNGQESELYTWKWKVIVAEQILALEHSGSG* | | | |
| *evm.TU.contig1.114* | | MENSTLESHHLRSSEHFVTYDSPYPLYAVALSPSAAHHRIAVGSFIEEYANHVDILSFDDDTLAIRPNPALSFDHTFPATKLMFRPSSAGKSADVLASSGEYLRLWEVRDSSVEQLLVLNNSKTSEFCAPLTSFDWNEVEPRRIGTSSIDTTCTIWDVEKGVIETQLIAHDKEVYDIAWGEAGVFASVSADGSVRIFDLRDKEHSTIIYESPQPDTPLLRLAWNKQDLRYMATILMDCNKVVILDIRSPTMPVAELERHRASVNAIAWAPQSCRHICSASDDTQALIWELPTVAGPNGIDPMSMYSAGSEINQLQWSAALPDWIAIAFSTKMQLLKV* | | | |
| *evm.TU.contig1.155* | | MQRGIPLSEEDRIPWLETLQGALRANLAIGKTCILGCSALQRKYREILRSADPEYETGSYGGGVKFVLLDAKADVLAARLKRRAEEGEHFMPATLLQSQLDLLDIDESEGILKVDATLSPAAIVNAIRDLACFCVFLLWILQVFSIHIMEAGSANSSSAGKTRSRYPLQEQLLQRRSSRENLDRFIPNRSAMDFDYAHYMLTEGRKGKENPAVSSPSREAYRKQLAETFNMNRTRILAFKNKPPTPSEAIPNEFSTIHQSKPTKSRRYIPQQTSERTLDAPDLVDDYYLNLLDWGSSNVLAIALGSTVYLWDATDGATSELVTIDDENGPVTSVKWAPDGRHIAIGLNNSDVQLWDSTSNRQLRTLRGGHQSRVGSLDWNSHILTTGGMDGQIINNDVRIRAHIIETYRGHQQEVCGLKWSASGQQLASGGNDNILHIWDRSVASSNSATQWLHRLEDHRAAVKALAWCPFQGNLLASGGGGGDRCIKFWNTHTGACLNSVDTGSQVCALLWNKNERELLSSHGFTQNQLTLWKYPSMVKVAELTGHTSRVLFMAQSPDGCTVASAAGDETLRFWNVFGAPEVAKPAPKANPEPFAHLNRIR* | | | |
| *evm.TU.contig8010.1* | | MGFLSSDCEMQFPWGKENTKHGTRENSLFVQTLLVLEKGSWDDFIPLEGGGQVHMNMQFVLSEEERNRIRSMRESAMKKKYGEQLSSGLGCSEMASSAGGILVSSSFANIEVSDGSEETSSTACVSKRRNVHLGEINHSKSVQQIGTWSFHLDDPERHVYFQDTPSITEKTSVASILKNWQADQLEKQEPLEKTPSNVRKVISAFESSLAKDMRVGMKPSPSKLHSDKVGPEAHKEVDLKKINQPGQLTSGRLKNPFLTGELQHIQVYKTLREDSHGSKFSQDSGQLKEPSAMQIEPMTKHKKPLEHLVRIPEVEATTVSQSMDKGRSFPANRLGSSSATFMEGNAIDIRATDSHEIYTLGSSEDKLKSAALFENDNLSSEIYDAWIFPDHAKRLCITTGSKQIMDLVGSCGIQERVHKSEKRCQFLENVEKFSSHKEVKKGGRTSPELRKSTFESSLEDETARGPMGQAIKVAIMIAFGTLVLLTRQRKPRHELQKMVRSIKNPKKAKRKNKGSKKGEGSSSSSSIPSLPAKVWQPGVDKIEEGEELQCDPSAYNSLHAFHIGWPCLSFDVLRDSLGLVRTEFPHTVYCVAGTQAEKVSWNSVEIFKISNISGKRRELVPSKSNADGSDMDSDSSDSDEDEVQDGGSSIPVLQLRKVAHEGCVNRIRAMSQNPHICASWADTGHVQVWDFSSHLNALAESETEASRGSSTVFSQAPLVKFGGHKDEGYAIDWSALVPGRLVSGDCKNCIHLWEPTSDTTWNVDANPFVGHTASVEDLQWSPTEPYIFASCSVDGNIAIWDIRSGKSPAASIKAHNADVNVISWNSRLASCMLASGCDDGTFSIHDLRLLKEGDSVVAHFEYHKHPITSIEWSPHEASTLAVSSSDNQLTIWDLSLERDEEEEAEFKAQTREEVNTPADLPPQLLFVHQGLKDLKELHWHSQIPGMLISTAADGFNILMPSNLESALPATNTA* | | | |
| *evm.TU.contig8949.159* | | MGEYEEAEIEQVEEEFAVWKKNTPFLYDLVICHPLEWPSLTVHWLPSPPSSHPSDDAFALHKLVLGTHTSEDFPNFLMVADAHIPRNPANFATDRPLDSAIPKVEIVQKIYVDGEVNRARCMPQNPSVVSAKTSGSEVYVFDCGKHPMTEEEGSCDPDMRLRGHEKEGYGLSWSPFKEGYLLSGSNDCKICFWDVSAMPQDKVLNAMHVYEAHESVVEHVSWHLKNENLFGSVGDDCLLAIWDLRTNKPQHSFQAHEKEVNYLSFNPYNEWIVATASSDTTVGLFDIRKLTTPLHIFSSHTEEVFQVEWDPNHETVLASSAGDRRLMVWDLNRIGDEQLEGEAEDGPPELLFSHGGHKAKISDFSWNKNEPWVISSVAEDNTLQVWEMAESIYHDEVQTMDCP* | | | |
| *evm.TU.contig4174.74* | | MTMENSSESGWQRSDSSRGFNASAVSDRNPRLFRASRIRSSSDASSHDSGFIPGGKERERNVLTNRDVRERSTSVSAVCKDEIAVHPVVCGVDWTDISLRQWLDNPERSVDAVECLHVFTQIVQIIELAHSQGIVVHNVRPSCFVISSFNSVSFLESSSCSDSGSDSLGDGSNSQTAEFKGSSSLLPRESHQQSSQFRRENSLRGIDPRSLYPTQNSFEAGSDVEQVEEKKQSFPMKQILLLESNWYTSPEEVGGAPSSCASDIYRLGVLLFELFCTFSSSEEKSSTMSSLRHRVLPPQLLLKWPKEASFCLWLLHPDPSSRPKMSDLLQSEFLNEPRNNIEERQAGIDLRERIEEQELLLEFLTLIQQRKQVAADNLQDTISFISSDIEEASKKQSIIRKKGRLCPEEGRDPSSGAALMEAVENEDSGCSGSRKRYRQGLHSYNAEKADDQLLEDQSSKASAGNQGCILLKSCRLLKNFKKLETAYFLTRRRAIKQSGKSSACHPPISAIGRGSITSNERNSNNNFPWNVQSSEERQSGWINSFFEGLCKYLSFSKLQVKADLKQGDLINSSNLVCSLSFDRDGELFATAGVNKKIKIFEYHRILNEDRDIHYPVVELASRSKLSSICWNSYIKSQIASSNFEGVVQVWDVTRSQVYMEMREHERRVWSVDFSLADPMMLASGSDDGSVKLWNINQGVSVGTVRTKANVCCVQFPINSGSSLAFGSADHRIYYYDLRNLKAPLCTLTGHNKTVSYVKFIDSMNLVSASTDNTIKLWDLSTCTSQISFTGHTNVKNFVGLSVSDGYIATGSETNEVYVYHKAFPMPASSFKFGSTDPFSGDDDAPQFISSVCWRGQTSSLVAANSTGNIKLLEMV* | | | |
| *evm.TU.contig3208.5* | | MESTPPRRKSGINLPATMSESSLRLEPLCGVSNLHSPRTMSPRTISNLSSSPAKSVTCSDRFIPCRSSSRLHTFGLVEKASPSKEGGCGGSGNGSEAYSRLLKSELFGSDFGCSVSSAGQASPMSPSRNMLRFKTDHSGPNSPYSPSILGHSGGFPSEPSTPPKPPRKVPKTPHKVLDAPSLQDDFYLNLVDWSSQNALAVGLGTCVYLWNATTSKVTKLCDLGPSDGVCSVQWTREGSYISIGTNLGQVQIWDGTQCKKVRTMGGHQTRTGVLAWSSRILSSGSRDRNILQHDLRVSNDFVSKLVGHKSEVCGLKWSHDDRELASGGNDNQLLVWNQHSQQPVLKLTEHTAAVKAIAWSPHQSSLLASGGGTADRCIRFWNTTNGNQLNSVDTGSQVCNLAWSKNVNELVSTHGYSQNQIMVWKYPSMAKVATLTGHSLRVLYLAMSPDGQTIVTGAGDETLRFWNVFPSMKTPTPVKDTGVWSLGRTHIR* | | | |
| *evm.TU.contig8026.189* | | MGFLSSDCEMQFPWGKENTKHGTRENSLFVQTLLVLEKGSWDDFIPLEGGGQVHMNMQFVLSEEERNRIRSMRESAMKKKYGEQLSSGLGCSEMASSAGGILVSSSFANIEVSDGSEETSSTACVSKRRNVHLGEINHSKSVQQIGTWSFHLDDPERHVYFQDTPSITEKTSVASILKNWQADQLEKQEPLEKTPSNVRKVISAFESSLAKDMRVGMKPSPSKLHSDKVGPEAHKEVDLKKINQPGQLTSGRLKNPFLTGELQHIQVYKTLREDSHGSKFSQDSGQLKEPSAMQIEPMTKHKKPLEHLVRIPEVEATTVSQSMDKGRSFPANRLGSSSATFMEGNAIDIRATDSHEIYTLGSSEDKLKSAALFENDNLSSEIYDAWIFPDHAKRLCITTGSKQIMDLVGSCGIQERVHKSEKRCQFLENVEKSWLKGWREASEKVANLAGMVLQDRGDRHELQKMVRSIKNPKKAKRKNKGSKKGEGSSSSSSIPSLPAKVWQPGVDKIEEGEELQCDPSAYNSLHAFHIGWPCLSFDVLRDSLGLVRTEFPHTVYCVAGTQAEKVSWNSVEIFKISNISGKRRELVPSKSNADGSDMDSDSSDSDEDEVQDGGSSIPVLQLRKVAHEGCVNRIRAMSQNPHICASWADTGHVQVWDFSSHLNALAESETEASRGSSTVFSQAPLVKFGGHKDEGYAIDWSALVPGRLVSGDCKNCIHLWEPTSDTTWNVDANPFVGHTASVEDLQWSPTEPYIFASCSVDGNIAIWDIRSGKSPAASIKAHNADVNVISWNSRLASCMLASGCDDGTFSIHDLRLLKEGDSVVAHFEYHKHPITSIEWSPHEASTLAVSSSDNQLTIWDLSLERDEEEEAEFKAQTREEVNTPADLPPQLLFVHQGLKDLKELHWHSQIPGMLISTAADGFNILMPSNLESALPATNTA* | | | |
| *evm.TU.contig2115.188* | | MGASSDPNQDGSDEQQRRSEIYTYEAPWHIYAMNWSVRRDKKYRLAIASVMEQCSNRVEIVQLDDSNGEIRSHPDLSFEHPYPPTQLIFIPDKECQKPDFFATSSDFLRLWHVVGEEGDQPHRVEMKSLLNNNRNSEFCGPLTSFDWNEAEPRRIGTSSIDTTCTIWDIERETVDTQLIAHDKEVYDIAWGGVGVFASVSADGSVRVFDLRDKEHSTIIYESSEPDTPLVRLGWNKQDPRYMATIIMDSAKVVVLDIRFPTLPVVELQRHQASVNAIAWAPHSSCHICTAGDDSQALIWDLSSMGQHIEGGLDPILAYTAGAEIEQLQWSSSQPDWVAIAFSTKLQILRV* | | | |
| *evm.TU.contig8011.2* | | MSHRVVQTLLVLEKGSWDDFIPLEGGGQVHMNMQFVLSEEERNRIRSMRESAMKKKYGEQLSSGLGCSEMASSAGGILVSSSFANIEVSDGSEETSSTACVSKRRNVHLGEINHSKSVQQIGTWSFHLDDPERHVYFQDTPSITEKTSVASILKNWQADQLEKQEPLEKTPSNVRKVISAFESSLAKDMRVGMKPSPSKLHSDEVGPEAHKEVDLKKINQPGKLTSGRLKNPFLTGELQHIQVYKTLREDSFGSKFSQDSGQLKEPSAMQIEPMTKHKKPLEHLVRIPEVEATTVSQSMDKGRSFPANRLGSSSATFMEGNAIDIRATDSHEIYTLGSSEDKLKSAALFENDNLSSEIYDAWIFPDHAKRLCITTGSKQIMDLVGSCGIQERVHKSEKRCQFLENVEKGSKKGEGSSSSSSIPSLPAKVWQPGVDKIEEGEELQCDPSAYNSLHAFHIGWPCLSFDVLRDSLGLVRTEFPHTVYCVAGTQAEKASWNSVEIFKISNISGKRRELVPSKSNADGSDMDSDSSDSDEDEVQDGGSSIPVLQLRKVAHEGCVNRIRAMSLNPHICASWADTGHVQVWDFSSHLNALAESETEASRGSSTVFNQAPLVKFGGHKDEGYAIDWSALVPGRLVSGDCKNCIHLWEPTSDTTWNVDANPFVGHTASVEDLQWSPTEPYIFASCSVDGNIAIWDIRSGKSPAASIKAHNADVNVISWNSRLASCMLASGCDDGTFSIRDLRLLKEGDSVVAHFEYHKHPITSIEWSPHEASTLAVSSSDNQLTIWDLSLERDEEEEAEFKAQTREEVNAPADLPPQLLFVHQGLKDLKELHWHSQIPGMLISTAADGFNILMPSNLESALPATNTA* | | | |
| *evm.TU.contig7281.264* | | MPVFKTPFNGYAVKFSPFYENRLAVATAQNFGILGNGRLHVLDLSPVPGRPISEFASFDTADGVYDVAWSESHDSLLVAASADGSVKLYDLSLPPTSNPVRSFHEHTRETHSVDYNPVRRDSFLTSSWDDTVKLWTIDRPASVRTFKEHAYCVYSVTWNPRHADVFASASGDCTARIWDVREPGSTMILPAHEFEILSCDWNKYDDCVIATASVDKSIKVWDVRSFRIPVAVLNGHTYAVRKLKFSPHRGSLIASCSYDMTVCLWDYMVEDALVGRYDHHTEFAVGVDMSVLVEGLLASTGWDELVYVWQNGTDPRAP* | | | |
| *evm.TU.contig1398.203* | | MAAPPSSRLQAMLQAAVQTVQWTYSLFWQMCPQQGILVWGDGYYNGAIKTRKTIQPMEVTAEEASLQRSQQLRELYDSLSAGESNQQTRRPCAALSPEDLTESEWFYLMCVSFTFPPGVGLPGKAYSKRQHVWLAGANEVDSKVFSRAILAKSARVQTVVCIPLLDGVVELGTTERVQEDIGFVQFVKNFFVDHHPPQPPKPALSEHSTSNPATSSEHARFHSSPLLPAMYAPVDPPADANQMEQEEEEDEEEEEEEEEEEDDEEDDEEEEEQEGGESESEANTGRNSRLSGGQNPHALSQGVGAAEAEPSELMQLEMSEDIRFGSPDDASNNLDSDFQLLAASQAGNPTDHQGRADSYRAESTRRWPVIVHDPLSTTSLQPPSSGEPALENLTHEDTHYSQTVSTILQHISSRWSESSSSATGYLMFSSQSAFSKWTGRPADHHFHVPIDGASQWLLKSILFSVPFLHSKNRDDNSPKSRYATDSTSRFRKGTPQDELSANHVLAERRRREKLNERFIILRSLVPFVTKMDKASILGDTIEYVKQLRSKIQDLEASARQMEMDQRSQRTNSLSLKEPRSGVTAVSDRSRSGGPPSGSDKRKLRIVEGTGGAVKQKVVNSPSQPPPPPPPPPPQPVPGVTTQVQVSIIESDALVELQCPHREGLLLDVMVVLREVRLEVTAVQSSLTNGIFVAELRAKVRENVGGKKPSIVEVKRAIHQKIH* | | | |
| *evm.TU.contig1398.106* | | MGTQLHQMLRTLCFNSEWKYAVFWKLDHQARTMLTWEDAYYDYHEQHEDPENMCSTEMGVSLHEWLLPDPLGLALAKMSYHVHSLGEGVIGHVAVTGRHMWIFADEHETDMSLPFEHFDGWQNQFSAGIKTVVLVAIFPHGVVQLGSLNKVPEVLKMLNHIRDIFYAHQHCLLGRSPSVGHSMLNSSSCLIDISSGSLVSGNFLDCISSIGRPVSKDDMQHTLSDHAYAVPLLGNLLKKTDEVTSKDRGIELSASWGAESAILVKPGSELFLLEQHEQVRKRLLIDRSWKWESSSLGYLGVGSEVQDTTSSVGSELYEICRPGFENETSNYNLEAENMENETATEMPEGMDSSSLLTVDSGSDNLLEAVVANACRGGNNVKIEKSESVKSTLTTGAMPLTTCNDQSLIEMETLHCLSSEAGGVSSSKGFLSRIHSTFDGQLDNAKEPAKVNRKRARPDENSRPRPRDRQLIQHRIKKLREIVPSGSKCSIDSLLERTIKHMLFMQSIIKRAAKLKPSTKPKLLSPEPVVRGLPRHDQGSTWAVELGSHLNFCPIMIENVNMNGQMLIEMMCEDCSHFLKIAEAIRSLGLIILKGTTKACGKKTRMCFVVEEDQNRSIHRLDILWTLVPILQSRASL* | | | |
| *evm.TU.contig1398.116* | | MGEKFWLNEEDKGVVEAVLGAEAFEYLVASASGNILSEFVPAAGDLGVQEGLCKVVEGSDWTYAIFWQVSKSKSGNSALIWGDGHCRGAKGVEAGGGDGNGSGDPESEKGDGKKKRVLQKLQACFGGSEEDNYAAGLDSVSDVEMFYLTSMYYAFPFDKPSSPSQSFNTGRVIWVSDAKSCFDHYQSRSFLANLARFETVVFVPTKSGVVEIGSVKSVPEEPTFVQMVKTTFAGVPLQAKVLPKIFGQELTLGSTNSKSMTLSFAPKLEDDVGFTSESYDLQAVGSGQVYGNCSNGSRGDDGDAKLFPQRNQILLGGLTSQAAVSGLEQSQEDLFVQQDDRKPRKRGRKPANGREEPLNHVEAERQRREKLNQRFYALRAVVPNISKMDKASLLGDAITYITDLQRKIKILEAEKEIVSNNQKPSSVPEIDFQARHEDAVVRVSCPLDAHPVSRVINTFREHQVMVQEAKVSTTDSGEVVHTFSIRTQAGAAEPLKEKLAAALSR* | | | |
| *evm.TU.contig26.15* | | MEKVMANYELIACGINGLPCDDCRKLGSREKEKRRVEDQTGRTWVVPRELDLSPNSPENALPPALSFSILSIFSLSLSLSLSGYNYLFPYNCFCFIRVQAMGYLLKEFLKTLCGVNQWSYAVFWKMADQNPKLLICEECYYEPTMPCYAVTCIPGTESSELAFEEWEAPRVSAKPRSARTQDQSEDKIGLLMDKMMMNNRFKVVGEGLVGRAAFTGNHLWILSENYARETQPPEVQNEVCHQFSSGIKTVAVIPVLSHGVVQLGSSSTIRENMAFVNNVKSLILELGCVPGDLLSVNFTSEELSPVVGIPVCLGKSALPDPSGSYKVEVSVPFEANTCNQQHISSQPVELLGQPSHSLIKQADGYLEGTSTFQAPKLAHNVVGSQIGLCQPNVNVRMKPSLLFSSHPAHRGTRGEIISSRPEILLNQQVYLQNLRPGLDEQPCVGSLFANSGYLGLMDEQFLSGALGENITNNLSTSSDCVTSQLRTNEEGLSSSCHPRSILNPCCLPMEVFLFDLMCHSTDNKFLSRSSNLIYHPGDDKCHQIEVAQKGGIENDLFQAHNIQLVDLGKNMNLSEDPSAFDHDSGKNKHGNTSPRSRNAEYQYECIQPSSRDDLFDIVGVDFKNKQFNDEWSAFCNSDMKTLGNNNCMPSNLQSADSDLYLVDGANSESGIFSATGTDHLLDAVVNRVHIAAKQSSDDSMSCRTTLTKISGSPIPSASPSNGLVSGSDERHGELFGLHTSLEKLGTAGSCSFKSESKKEDAGNGLNTSSIYGSQISSWVEQGHSSKQNNSASTAYSKRPDEVNKPSRKRLKPGENPRPRPKDRQMIQDRMKELREIVPNGAKCSQDLLLERTIKHMLFLQSVTKHSDKLKQVGESKILDKDGGLLLKDNCNRGRTWAYEVGSQSVVCPLIVEDLNSPRQMLVEMLCEERGLFLEIADVIRGLGLTILKGVMETRNDKIWAHFAVEANRDVTRMEIFLSLIHLLEQNVQSSVAPGNGIENGDMAVHQNHAALIPATGGCSSQ* | | | |
| *evm.TU.contig1399.135* | | MTDYRLPMNPWTDDNASMTEAFMSSSDPTSFWHPPSSSTTAAAEPSKVINQAPPLPPAQFNQETLQRRLQALIDGGRESWTYAIFWQSSMVGYSGGSMLGWGDGYYKGDEGKGKRKSSSSSSAAEQEHRKKVLRELNSLISGASPSADDSVDEEVTDTEWFFLVSMTQSFINGSGLPGQAFLNSTSIWVAGAERLAVSQCERAQQGQVFGLQTLVCIPAANGVVELGSTELIFQSSDLMNKVRVLFNFNGVETGGPWSLQMDHGENDPSALYITEPVSEARDSLNTATVIPSNNPPLSKQTTITENPSSIHHQNQPQQSLFSRELINFSEFGYDGSSVRNGNSHSCKPESGEILNFGGSKRSSNGNVLSGHSQFGSGDDEKKKRSPPSRGSNEEGMLSFSSGVIVPSSGVVKSSGGVDSDHSDLEASVAREADSSRVVEPEKRPRKRGRKPANGREEPLNHVEAERQRREKLNQRFYSLRAVVPNVSKMDKASLLGDAISYINELKSKLHAAESDKEEMRSKIESIQKELAVKDSRFPGPPPPSHDLKMPNQHGGKLIDMDIDVKIIGWDAMIRIQCGKRNHPAARLMSALKELDLDLHHASVSVVNDLMIQQATVRMGSRFYTQEQLRLALSARLADAR* | | | |
| *evm.TU.contig1618.21* | | MDEYSSIISAASSSPSSIQLQSPATLQQRLQLIVQTQPHCWAYAIFWQSFNDDINGRLVLAWADGHFHETTKHTVLHTDRSKPIVGEANDAVEWFFLMSSTRSFCAGDGVPGKAFSSGSLVWLKAAHQLQFYNCERARDAHAHGLLTLVCIPTCNGVIELGSTQIIQENWGLVQQVKSLFGSDLPIFPKQLMSPMQVLDHRNVSVADVGIVMEHSKSECGLEPPPAEAMQQRQPKKRGRKPGQGRETPADHVEAERQRREKLNHRFYALRAVVPNVSRMDKASLLSDAVAYITELKGKVEDMASKLHQRESSSKNVKKMESIDSSADNQSTTTTSVDQRTGPNSSSSSSSPRCTAAGLEVEVKIVGPNAMVRVQSENVDYPAARLMVALQELELPVHHASISTVNDLMLQDLVVGAPEGLSEDGFRTALLGKLNYSSKSNSLI* | | | |
| *evm.TU.contig8910.528* | | MDELVISCFSSSSVVSPQENPPSNLQRRLHFILQNQPEWWIYAIFWQPSDDGNDSLVLAWADGHFQGTKRPLPNADNQRKLVRGNLQATISTETAQIIDEPLDAQLFYLMSSTRSFSAGEGVPGRAFSSGSFVWLTGAHQLRFYDCERAKEAETHGIETLVCIPTACGVLELGSNDMIKENWALVQQAISLFRSDPIVSKQPNPTSKVLIQFLDERNINSFADTGIVAGLQEEDFIDDIVDDKLLDGDAKTATTKVEAAIDAAGNESEQSELQQQQQRVPKKRAQKAAGAKPLNHHVEAERQRRERLNSRFYALRSVVPNVSKMDKASLLSDAVSYISQLKAKVEDLESQLLHHTVAGSKRLKTEAADTAENQSAATTNTTSTCVEENQAGILPTSPPSPSSSGGPVVPLEIEVKVVGGDAMIRVQSENAEYPTARLMEALRDLQLPVHHASISTVNYLMLQDIVIRAPHEGSLKTEEGLKATLLRRLSQRC* | | | |
| *evm.TU.contig8910.303* | | MANRVQNQEGVPENLRRQLAVAVRSVQWSYAIFWSLSSRQEGVLEWRGGYYNGDIKTRKMVQGMELKPDRMGLQRSEQLRELYECLLEGEPDQQPKKASAALSPDDLTDVEWYYLVCMSFVFDPGQGLPGRSLATGQPIWLCNAHSADSKVFSRSLLAKSASIQTVICFPHFRGVIELGVTELVPEDPSLIQHIKVSLLEFSKPICSEKSSSAPYSGDDDTDCMYTKFDHEIANTTVLDNFHSPRKDTRSEEEKINKFNGNIENDLTVSSPDDCSNGCEHHHQTQDSFLHEGINGGSSQVQSQHFRDDDFSNCDQDSMNSSNCISQPFVNQENVFSPTKGDYMDKIHLEEPQECNYMKSSSLDLATHGDLYYARTLTNILRNSHWLLENLRFGSNDYKSSFVSWKKGEIAKSHWPEVQQKMLKKILFLIPLLQGSCSFKSQKVNFTKDWLLKPMSGEICMRRPFSYERTENEKFLALRSMIPPISKIDKVSILDNTIEYLRQLEARLEELESFIDFGERKTEATRKHPDLVEQTCENTEDRRIGNGNNWFNKRKACDIDETDPGLSSVVPKAGPLLDMKVKIKEQDVVIELRCQWRDYLLLDIIDTLNNLNLDVFSVQSSTTDDVLNVTLQAKFRGAAVASARMIKQTLSTIAGKC* | | | |
| *evm.TU.contig8910.474* | | MKVEMGAAGGGVWSDEDKAMAAAVLGTRAFDYLMSSLVSSEGSLVAIGSDENLQNKLSDLVDRPNAANFSWNYAIFWQISRSRTGDLVLGWGDGSCREPKEGEESEVLRILNFRLEDETQQRMRKRVLQKLHTLSGGLDEDGYAFGLDRVTDTEMFFLASMYFLFPRGEGGPGKCFASGKHVWLSDILKTSDEYCVRSFLAKSSGIQTVVLIPTDIGVVELGSVRSIPESLELVRSMRSSFSSFSSLLRAKTIAATPVLSEKKEGNAHFLNVRVGDGVPKIFGQDLNMVRSQFREKLAVRKAEERPWEAYPNGGQNPFTNARNGLHGSGWTQFHGGKQSSVLEIYSPQTPVNNPPGLVNGFREDFRLKNYLPPKSLPLQIDFTGGATSRPCTMPRPGSVESEHSDVEASVKDEQADQADGKRPRKRGRKPANGREEALNHVEAERQRREKLNQRFYALRAVVPNISKMDKASLLGDAIAYITELQKKLKDMESEREKLGSTSRESFPEEASNPTTENRNQVPEIKIEAAAPDEVIVRVNCHLDTHPVSRVIRAFKEAQITVVDSKLATGKDTVFHTFVIKSQGLEQVTKERLIAAFSGESNSLQP* | | | |
| *evm.TU.contig8026.432* | | MGYLLKEVLKTLCGVNQWSYAVFWKIGYQNPELLIWEECYYESIPSPTLPPIAAIGSSGLALEEWEASCVSAEHRNLLAIARPDVLVPSLVDKMMMCSPIKVVGQGLVGRAAFTGKYLWVLSENYAREAYPPEVQNEVCQQFSAGIKTVAIIPILGHGVVQLGSSLAIMENVAFVNTVKSLMLQLGCVPGALLSDNYTAKEHPPMIGAAVFPEKSISADPSGNYKVKDSVPSVADGSYSCYQQSIPSQAVGSHFTFKQIENNLHGNSSTFQTPNLTQNIARSSLNGPCLPNIIPGTKPGRLLSSLLEIEETRAEVISLNPHTRLSQQESLYNHRSKFGEQPPVGPSSTNYSNSRINDAQRLPNAVLQERANNYLSNSCGIRMSQQVSANGNQSSRAGQLQTGGSDPRSSPNLCSLLNVHKSAGIDVSCTCLPGIGINAGPSETEVSTSKPMDHLTPNHLLLQSTGFRRHSHIDEGSQTELPQRNGTIENNLFQALNILSTPLDEQMSLSEKFYGLDYNSQKPEYGNESSRSKSATNENEYAQPPSGDDLFDILGMDFKKKVFSDSWNNFYNHEPDTNKNTSIDDMVTSLNPRDADPDLYSFDGGNSDSGIFSVTGTDHLLDAVVNKARIALNQSSDESASCKTTLTKINSSSVPIASPSYGQVSISDQKQGEFCGLPKPLAKLAPVGSCSFKSECNKDTKSYSQANSVYGSQINSWVEQGDSTKHNNSASTACSKRPDEVSKLNRKRLKPGENPRPRPKDRQMIQDRLKELREIVPNGAKCSIDGLLERTIKHMLFLQGVTKHADKLKQIGESKIVSKDPGMLLGHNFEGGRTWAYEVGSQSMVCPIIVEDLNAPRQMLVKMLCEERGLFLEIADMIKGLGLTILKGVMETLNDKVWARFAVEANRDVTRVEVFLSLVHLLEKTGKRSAASGNGAHDDNMNTVQQHFPQAAPVSASGRSCSLH* | | | |
| *evm.TU.contig5830.25* | | MDYRVPENLRRHLAYAVRSIQWSYAIFWAISSRHQGMLKWGEGYYNGDIKTRKTVQAMEVNEDQLGLERSEQLRELYESLSAGESSSSPQSRRPSAALSPEDLTNTEWFYLVCMSFFFNIGQGLPGRTLANGQPIWLCNAHYADSRVFSRSLLAKTVACFPFSGGVIEIGVTELVPEDLGLIQHIRTSFLGIPCPVPQISTYSKPNQEILHTNFDAIVECEDVKVCSPHSSSNRFGLTQQAEELLMADDLNEGASQVQSWQLMDDEVSYCDHNSMSSIDCISQTWVNCEKVREKENENDGFLLDIQEDNQMKLTSMDLQADDIHYHTVVSTLLKSSHQLILGPNFRKLHHKSSFVRWKKGGFEDTLLPQSGAASQRLLKKVLFEVARMPGGCWVESREDYGGSDGLWRPQVDSVESTHALAERRRRGKLNEKFVVLGSLVPSTSKADKASILDNAIEYVKELKRKVEELNSFREPANQEARTKRKPQDITERTSDNYGDNSNSNSKRFLTNKHKATNCIVVEDEDNSTDNVTVSMAEKGVLIEIRCPWREWLLLEIMNAISRLHLDPSSVQSTNIDGILSLTIKSKFKGSAVGSLRIIRQALQRAVEECGSNNRNPAAF* | | | |
| *evm.TU.contig3039.240* | | MHAKSSIGTIRKALDDQNGQTNRKSNRTGQRGRAAMYTETSARDEAAAAMKALATTLQRLRPLVQSRAWDYCVVWQLGNDPSRHCFFLFWFIKWMGCCCNGGYGDHPSASMVNEERAREEEDRLVPLCRDLFSRHPAKTSACVALSRFPPSIPLYSGVHGEAVISMEAKWLSCTEELLNSNKCHESTGTRVLIPVVGGLIELFVAKQIPRDQKIIDLVADQCKITMAQKATSAQDHSAMRLNAQPHDAEYSNNRLSHLQYLNLFPGLPIHSRVSQPGAYPVLEGSPASSNPSNEHFSMDSCSGYPSPNVSVSETTRKHPILKRPISSRDMPVKQEFGFLVESTPNHLVERDNLKSRLSLEREQHQSKNLITERNRRKRIRNAHFALRALVPKITKMDTASILKDAIEYIEELQEEEKKLVDELREMAKEESDNKNDHPNIPEAGAYKGTGNSNATMQNKDRSSVDEKKHMEVQVEVNQIGARDFLLKCLCKKKQGGFLRLMEAVDALGLQVIDANITAFNGKVLNILRVEVNNMEIQPKTLKDSVIKSCTSRSSRQSSMENP* | | | |
| *evm.TU.contig5824.42* | | MTDYRLATMNLWADENASMMMEAFMASDLPSFWPPPSSSTTAAAAEPSKAIHHQSLPPQPVAVFNQESLQQQLQALIEGARESWTYAIFWQSSVDHAGGSILGWGDGYYKGEADKAKRKTTTSPSEQEHRKKVLRELNSLISDSSPSADDTVDEEVTDTEWFFLVSMTQSFPNGGGLPGQAFFNSTPIWLAGADSLACSLCERARQGQVFGIQTMVCIPSFNGVVELGSSELIFQSSDLMNKVRVLFNFNSIESASWSMQTDHSENDPSALYITDPSSAEIRDSVNTAKQITFENPPVIHEESQGHSQSTYTRELNFAEFVNEGSSTGNGNSRPSKPESGEILNFGESKRSSNQSHFGIAEEKKKKRAPTSRSTIEEGILSFSSGVVLPCSTATGEESNQADLMGSAARDADCRRVVDPEKKQRKRGRKPANGREEPLNHVEAERQRREKLNQRFYALRAVVPNVSKMDKASLLGDAISYINELKSKVQAAESDKEEMSSQIERLKKEMAAKESRNSSPPPRLDPDPKHPSHHQNSLIDTEIDVKIIGWEVMIRMQCSKKNHPAARLMATLKELDLDVQHASVSVMNDLMIQQATVRMGSRFYTQEQLRLVLLSHLADS* | | | |
